# Preferences, attitudes and views regarding genetic newborn screening (gNBS) for rare diseases: a systematic review of the literature and synthesis from 2009 to 2022

**DOI:** 10.1186/s13023-025-04179-0

**Published:** 2026-01-08

**Authors:** Sylvia Martin, Gergana Kyosovska-Peshtenska, Jennifer Audi, Kaja Zarakowska, Åsa Grauman, Jorien Veldwijk, Brett Hauber, Joshua Coulter, Aileen Fürer, Alexandra Wagner, Aneta Piperkova, Edith Sky Gross, Ferdinand Knieling, Gulcin Gumus, Marek Zak, Maria Martinez-Fresno, Alicia Granados, Stefaan Sansen, Yuen Man, Janbernd Kirschner, Lucia Pia Bruno, Enrico Silvio Bertini, Silvia Ottombrino, Antonio Novelli, Emanuele Agolini, Sandra Courbier, Nicolas Garnier, Tsungai Jackson, Branimir Velinov, Jessie Dubief, Roman Raming, Christina Saier, Fernanda Fortunato, Vera Frankova, Mats Hansson

**Affiliations:** 1 Center for Research and Bioethics (CRB), Uppsala University, Husargatan 3, BMC, Entrance A11, Uppsala, Sweden; 2 Bulgarian Association for Personalised Medicine, 45 Bacho Kiro str, Sofia, 1202 Bulgaria; 3 UCB, Allée de la Recherche, 60, Brussels, 1070 Belgium; 4 Pfizer, 66 Hudson Blvd235 E 42nd St, New York, USA; 5 Takeda Pharmaceuticals International AG, Zurich, Switzerland; 6 Novo Nordisk, Novo Alle 1, Bagsværd, 2880 Denmark; 7 Illumina, 5200 Illumina Way, San Diego, USA; 8 Sanofi, Diegem, Barcelona, Belgium; 9 Department of Neuropediatrics and Muscle Disorders, Faculty of Medicine, Medical Center – University of Freiburg, Freiburg, Germany; 10 Siena University, Telethon Institute of Genetics and Medicine (TIGEM), Sienna, Pozzuoli, Naples 80078 Italy; 11 Research Unit of Neuromuscular and Neurodegenerative Disease, Ospedale Pediatrico Bambino Gesu, IRCCS, Roma, Italy; 12 EURORDIS, Plateforme Maladies Rares, 96, rue Didot, Paris, 75014 France; 13 Department of Pediatrics and Adolescent Medicine, Universitätsklinikum Erlangen, Friedrich-Alexander-Universität (FAU) Erlangen-Nürnberg, Erlangen, Germany; 14 Erasmus School of Health Policy & Management, Erasmus University Rotterdam, P.O.Box 1738, Rotterdam, 3000 DR The Netherlands; 15 The Comparative Health Outcomes, Policy, and Economics (CHOICE) Institute, University of Washington School of Pharmacy, Seattle, WA USA; 16 University of Ferrara, Ferrara, Italy; 17 Charles University - First Faculty of Medicine, Prague, Czech Republic; 18 Laboratory of Medical Genetics, Translational Cytogenomics Research Unit, Bambino Gesù Children Hospital IRCCS, Rome, Italy

**Keywords:** Genetic newborn screening, Rare diseases, Preferences, Attitudes, Parents, Healthcare, Decision making, Ethics

## Abstract

**Background:**

Newborn screening (NBS) and its genetic version, genetic NBS (gNBS), are now used to identify a broad range of conditions, including metabolic, endocrine, and genetic disorders, leading to significant reductions in infant mortality and long-term complications. Advances in genomic technologies, particularly next-generation sequencing, have enhanced the ability to detect rare diseases early, using gNBS, improving long-term outcomes. The availability and scope of gNBS vary across countries, influenced by national policies and technological advancements. This systematic literature review aims to clarify the specific barriers, opportunities, and more general attitudes that stakeholders express about gNBS for rare diseases.

**Main:**

We extracted articles from 2010 to 2022. We followed the PRISMA guidelines and registered the review via PROSPERO (CRD42022297678). From an initial retrieval of 4519 records, two selection rounds resulted in a final list of 112 articles, which were assessed across different categories exploring various aspects of gNBS. The most important perceived opportunities in gNBS were the benefits of early intervention to reduce the burden of the diagnostic odyssey. The main identified barriers included three key codes: the stress and risk associated with false results and dealing with uncertainty (*n* = 25), the psychosocial implications (*n* = 26), and misunderstandings due to lack of education or communication. The majority of respondents expressed positive views, particularly regarding actionability.

**Conclusion:**

The results indicate a generally favourable attitude toward newborn screening, with subtle variations in viewpoints. Our findings on these themes can specifically inform how final attitudes are shaped based on particular aspects.

**Supplementary information:**

The online version contains supplementary material available at 10.1186/s13023-025-04179-0.

## General introduction

Newborn screening (NBS) was introduced in the 1960s with the successful implementation of the first screening programmes for phenylketonuria [1] and has since progressed toward genetic newborn screening (gNBS) techniques with the introduction of mass spectrometry [2]. Since then, gNBS has enabled early detection of a range of metabolic, endocrine, haematological, immune, cardiac, and pulmonary disorders, allowing early diagnosis and intervention in treatable diseases, thereby reducing disease progression, complications, and infant mortality [3]. Timely diagnosis and treatment can be particularly beneficial in rare diseases (RDs) [4–7]. As a standard public healthcare measure in developed countries, gNBS was recognised as one of the top ten public health achievements of the 2000s [8], benefiting more than 100,000 children worldwide [9].

About 80% of rare diseases (RDs) are genetic and can be diagnosed neonatally using genomic technologies, with improvements in sequencing enhancing accuracy and speed while reducing turnaround time [10] and costs [11]. This now enables the detection of disease before the appearance of symptoms, allowing timely prevention [12]. However, the benefits of gNBS for RDs without available treatment are less straightforward [13]. Over a decade ago, the ACMG recommended reporting pathogenic secondary findings associated with “actionable” disorders across all ages, arguing that it is in the patient’s best interest due to potentially preventable health outcomes [14]. This recommendation, however, raised concerns regarding what constitutes “treatable” [15] or “actionable disorder” [16, 17].

Screening always requires communicating potential benefits, such as life-changing treatments, and potential harms, including psychosocial impacts and implications for family planning decisions. Appropriate genetic counselling and education can help mitigate parental emotional distress. [18].

However, the complexity of information, uncertainty of the results, and misunderstandings about efficacy can lead parents to refuse gNBS [19]. Parents rely on support from clinicians and medical genetic counsellors to obtain reliable and complete information about the severity of the condition and its effect on their child’s life [20]. In contrast, healthcare providers (HCPs) appear to favour selective disclosure, protecting the child’s autonomy and right to access information after reaching adulthood. Nevertheless, both sides emphasise the need for a carefully planned approach to the integration of gNBS into healthcare practice [21].

Optimising the screening process seeks to minimise family distress caused by false positives or anxiety-inducing results, while ensuring reliable diagnosis of true positives [22], thereby ensuring continuous improvement in both the specificity and sensitivity of testing [23].

Despite these clear advantages, gNBS programme availability and implementation vary widely across Europe, and increasing genomic testing potential will likely amplify these differences [24]. European Union countries adopt different approaches toward the implementation of gNBS, which is influenced by decisions taken by national authorities regarding which diseases to add to the national panel [25–27]. All advances in gNBS techniques (e.g. whole-genome sequencing) can raise ethical and societal concerns, and policymakers must balance the rights and needs of the child with those of the family in gNBS [28].

This systematic review aims to identify the factors that influence acceptance of gNBS for rare diseases (RDs), including the preferences and perceptions of different stakeholders regarding gNBS.

## Methods

Experts from different backgrounds (medical geneticists, public health researchers, clinical psychologists, ethicists, and preferences studies researchers), developed a search strategy in collaboration with Uppsala University (UU) Library services. In January 2022, the review was registered in PROSPERO (CRD42022297678). Databases were selected from both the social sciences and medical fields (PsycInfo, PubMed, Scopus, and Web of Science; full search strategies are provided in Supplementary Document 1). The first concept represented the target population for genetic screening (Infant, Newborn [MeSH], babies, baby, etc.), the second targeted the techniques (Genetic Testing, Neonatal Screening, etc.), the third included terms referring to attitudes and experiences (Attitude, Emotions, opinion*, perception*, preference*), and the fourth targeted participants (Family, Nurses, Parents, etc.). The timeline for the search was from 2009 to 2022.

**Fig. 1:**
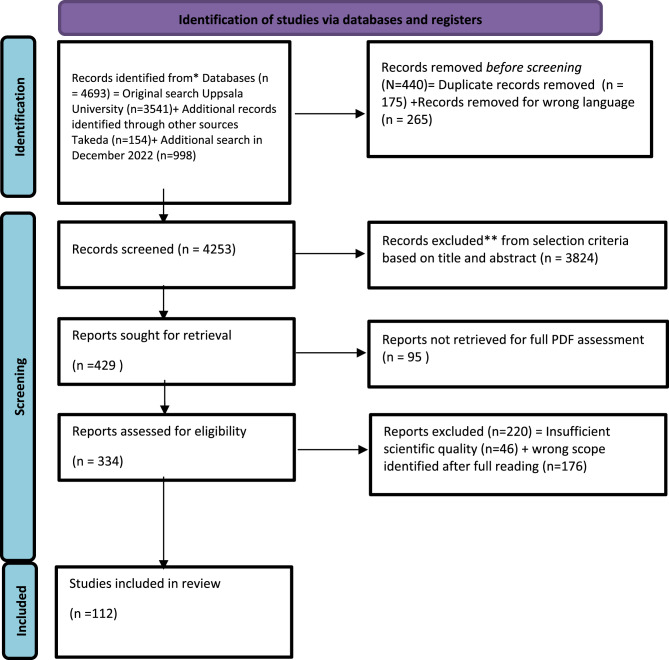
Prisma chart. *consider, if feasible to do so, reporting the number of records identified from each database or register searched (rather than the total number across all databases/registers). **if automation tools were used, indicate how many records were excluded by a human and how many were excluded by automation tools. *from:* page MJ, McKenzie je, bossuyt pm, Boutron I, Hoffmann TC, Mulrow cd, et al. The PRISMA 2020 statement: an updated guideline for reporting systematic reviews. BMJ 2021;372:n71. doi: 10.1136/bmj.N71. For more information, visit: http://www.prisma-statement.org/

The first search was performed in January 2022, yielding 3695 articles. This total comprised 3541 articles directly retrieved from the UU search and 154 articles suggested for examination by experts on rare diseases from the pharmaceutical company Takeda. An updated search was conducted in November 2022 to capture newly published papers between January and November 2022, resulting in 978 additional articles. Using the Rayyan.ai software, all articles were double-blinded and reviewed for the first selection based on title and abstract, followed by 334 full-text articles assessed for inclusion and exclusion (see Fig. 1, PRISMA chart; final selection criteria are presented in Supplementary File 3).

**Fig. 2:**
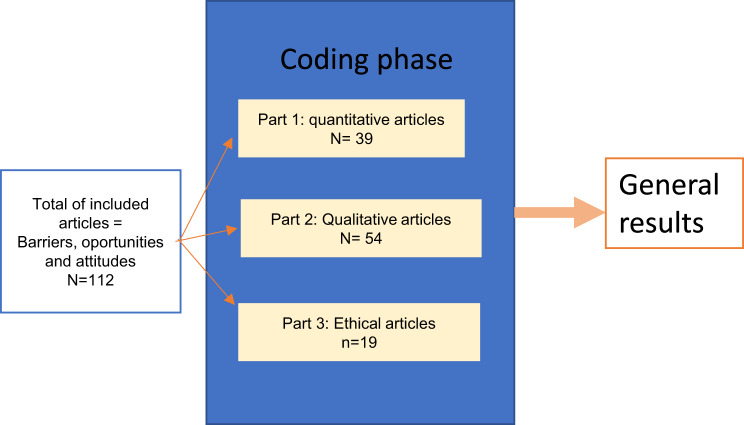
Analysis flowchart. Note: we differentiated them by the three series of outputs using lucid differentiation of articles based on methods: “quantitative” (cohort studies, analytical cross-sectional study, prevalence studies, RCTs, and systematic literature review & review synthesis) for part 1 and “qualitative” (case-control studies, case reports, case series, qualitative research, and text & opinion papers) for part 2 and part 3. For differencing qualitative articles from ethical articles the selection was made on the basis of the core topic explored with part 3 including the ones referring to policy-making and ethics articles based on the literature or as a review (excluding survey and questionnaire-based articles). This method complies with Cochrane’s guidelines recommendation to separate the analysis into different sub-analysis when the final number of articles is above 50, allowing a specified and targeted analysis of the content for each individual paper [29]

In the next step, full-text reading of the included articles was completed by separate teams of two reviewers or experts (11 pairs in total, one from the Social Science and Humanities–Public Health domain and the other from a medical background, such as a clinician, diagnostic specialist, or medical geneticist/genetics expert), with each pair assessing 30 to 31 articles. Final decisions on inclusion and exclusion were reached by consensus under the lead of the UU team. Scientific quality assessment (appraisal scoring) was carried out by an external company (LUCID Ltd, Marlow, England) using the JBI tools (Joanna Briggs Institute Model of Evidence-Based Healthcare) (see Table 1: Type of paper and Quality score). Only articles with sufficient quality (above the mean for their category) were included in the analysis (see Fig. 1, PRISMA chart). The final list of included articles comprised 112 papers, which were regrouped into different categories for refined analysis (see Fig. 2: Analysis flowchart): quantitative, qualitative, and ethical studies.

**Table 1:** Type of paper and quality score

					Score Range			Mean		
Category	No. Articles	No. Articles INCLUDED after selection	No. Articles EXCLUDED after selection	Max. Score	Low	High	Range	Score	%	Cut-off
Analytical Cross-Sectional Studies	2	1	1	6	5	6	1	**5.50**	92%	**6**
Case Control Studies	2	1	1	10	8	10	2	**9.00**	90%	**9**
Case Reports	1	1	0	7	7	7	0	**7.00**	100%	**7**
Case Series	2	2	0	8	8	8	0	**8.00**	100%	**8**
Cohort Studies	55	34	21	10	7	10	3	**8.51**	85%	**9**
Prevalence Studies	1	1	0	7	7	7	0	**7.00**	100%	**7**
Qualitative Research	66	48	18	10	7	10	3	**8.14**	81%	**8**
RCTs	4	3	1	9	8	9	1	**8.75**	97%	**9**
Systematic Review and Research Synthesis	9	5	4	10	6	10	4	**7.44**	74%	**7**
Text and Opinion	53	53	0	6	5	6	1	**5.32**	89%	**5**
	**195**	**149**	**46**

We employed thematic analysis to thoroughly investigate the selected articles. Thematic analysis involves identifying recurring patterns (themes) within the data and does not rely on any pre-existing theoretical framework. This approach allows for a combination of deductive, concept-driven methods, commonly used to establish main categories, and inductive, data-driven methods to identify subcategories [30, 31]. The data were structured into overarching themes and subthemes. The three overarching themes were: 1) Barriers (elements present that act as obstacles, flaws, or risks), 2) Opportunities (elements representing benefits and advantages), and 3) Attitudes (overall opinions or perspectives expressed in the articles, including the prevalence of barriers and opportunities).

After dividing the articles into the three categories, each section was reviewed by two independent coders (Part 1: GKP, SM; Part 2: GKP, JA; Part 3: SM, JA) to identify themes. Each reviewer proposed themes, which were then merged upon reaching consensus. To verify the suitability of the thematic system, the reviewers cross-checked their colleagues’ ratings a posteriori, ensuring that all themes were applied and were relevant to the aims and scope of the papers.

**Barriers** to acceptance were divided into the following subthemes: 1) Impact on intra-familial relationships, 2) Consequences of carrier status, 3) Misunderstanding of shared information and education, 4) No actionability^1^, 5) Burden of costs and logistics, 6) Discrimination and privacy, 7) Diagnostic uncertainty (including false-positive stress), 8) Not discussed, not captured, or not applicable (N/A), 9) Religious beliefs, and 10) Psychosocial implications.

**Opportunities** were divided into the following subthemes: 1) Early diagnosis and early treatment, 2) Family planning, 3) Understanding the future, 4) Improved healthcare and scientific advancement, 5) Improved quality of life (QoL), and 6) Not discussed, not captured, or not applicable (N/A).

**Attitudes** were divided into the following subthemes: 1) Positive attitudes from early diagnosis and early understanding, 2) Positive attitudes strictly related to actionability, 3) Attitudes varying with information and education, 4) Not discussed, not captured, or not applicable (N/A), 5) Negative attitudes, 6) Mixed attitudes (both positive and negative judgements), 7) Support strictly among parents and the general public, and 8) Support among healthcare providers (HCPs).

Five meta-themes were identified: A) Psychological implications, B) Education is key for those who provide and those who receive it, C) “Actionability” potential is crucial for positive perception, D) Ethics and clinical utility, and E) Differentiating impacts: focusing on one rare disease or many.

## Results

### Descriptive analysis

**Fig. 3:**
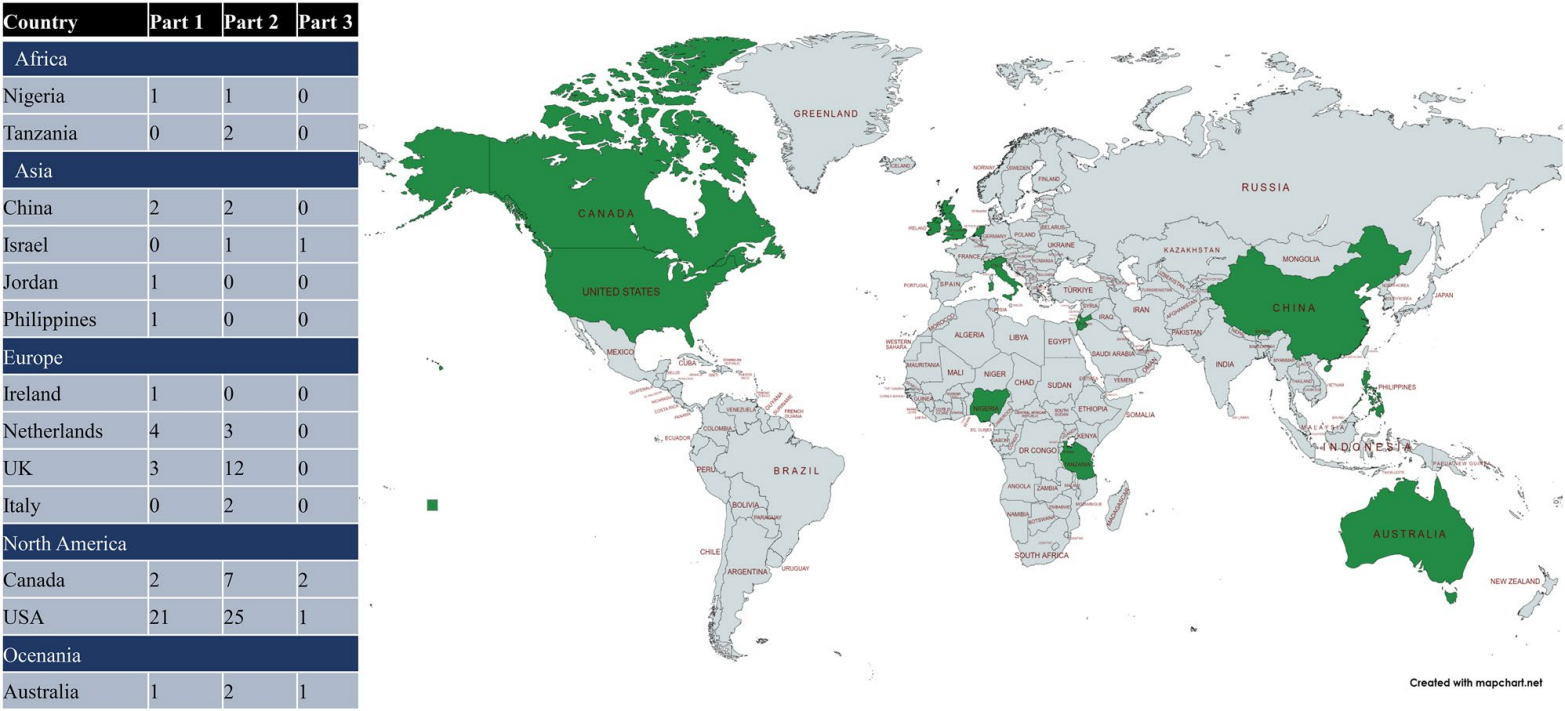
World repartition

For part 1 – quantitative studies, we extracted 40 articles (see Appendix 4). Most of the articles were cohort studies (32 cohort studies, 3 randomised controlled trials (RCTs), 4 SLRs, and one analytical cross-sectional study) and represented data from different regions of the world (see Fig. 3: World repartition). Approximately one third (16/40) of the articles did not focus on specific RDs or conditions (when more than one condition was assessed).

For part 2, we extracted 56 articles (see Appendix 4). Most (27/56) did not focus on a single specific RD. Over time, their methodology shifted towards more interviews and fewer surveys, with limited use of focus groups.

For part 3, we extracted 19 articles (see Appendix 4). Most of the articles were published between 2011 and 2015 (*n* = 3), but at least one article was published each year on the specific topic of ethics in gNBS/NBS. Seventeen articles were classified as “Text and opinion.” Most of the selected articles (8/19) were not disease-specific.

### Subthemes results

The results for all subthemes are presented in Appendix 5. The most important perceived opportunities in gNBS were early intervention to reduce the burden of the diagnostic odyssey (*n* = 21), followed by improving healthcare (*n* = 15). The least frequently reported opportunities were family planning (*n* = 11), supporting advances in knowledge and science (*n* = 8), and obtaining information and understanding (*n* = 7).

For barriers to gNBS, the three main codes were stress from the risk of false results and dealing with uncertainty (*n* = 25), psychosocial implications (*n* = 26), and misunderstanding due to lack of education or communication (*n* = 25). Other noted barriers included the burden of being part of a study (*n* = 13), impact on intra-familial relationships (*n* = 12), fear of discrimination (*n* = 10), consequences of carrier status (*n* = 9), and lack of actionability (*n* = 7). Religious beliefs were cited in only two papers.

Regarding attitudes, most of the respondents were positive, relating to traditional positive motives (*n* = 14) but also to actionability (*n* = 12). Mixed and negative attitudes were almost equally represented (*n* = 5 and *n* = 4, respectively).

Qualitative research findings support quantitative results regarding the observed determinants of gNBS uptake (see Appendix 5). Similar to the positive attitudes toward gNBS found in quantitative results [32–35], qualitative research extends these findings by providing a deeper interpretation and identifying cause-and-effect relationships that influence the decision-making process for gNBS. Unlike quantitative articles, which primarily express positive attitudes numerically, qualitative observations enrich the research hypotheses through interpretative analysis. Opportunities in qualitative articles were predominantly associated with the potential for early treatment and diagnosis (*n* = 27), with family planning arguments holding relative importance (*n* = 20). In these articles, the three main barriers identified were misunderstanding of information and limited education (*n* = 34), followed by psychosocial implications (*n* = 30), and the risk of false results leading to stress (*n* = 23). These findings align with those of the quantitative articles but show a relatively greater emphasis on logistical burdens (see Appendix 5).

In the analysis of ethical papers, the most important themes were early diagnosis (*n* = 12) and family planning (*n* = 12), followed by improved healthcare (*n* = 8) and improved quality of life (*n* = 7). This contrasts with research-based articles (Parts 1 and 2), which emphasised different priorities (see Appendix 5).

The first noted barrier was psychosocial implications (*n* = 17), followed by the risk of false results or uncertainty (*n* = 16), the consequences of carrier status (*n* = 12), and misunderstanding (*n* = 13). Concerns about privacy and the burden of participation were also significant.

### Meta-themes results

#### Psychological implications

##### Methodological issues in clearly measuring psychological aspects

Based on the thematic analysis in Part 1 (from Barriers, theme 7, *n* = 25, and 10, *n* = 26, with *n* = 15 including both themes), we specifically examined emotional and psychological issues. In ten papers, the measurement of psychological aspects was based on a clinical scale or validated instrument, whereas the majority (*n* = 15) assessed these issues through more qualitative methods, such as researcher-created questions or interview guides. Across these articles, seven focused on expected emotional or psychological issues rather than on actual measured scores (see Supplementary Document 2 for all scales used).

##### Anxiety: the primary psychological aspect

In the definition of “psychological aspects”, anxiety was the main element (*n* = 8), followed by stress (*n* = 5), worry (*n* = 2), depression (*n* = 2), burden (*n* = 2), guilt (*n* = 2), hypervigilance (*n* = 1), feeling overwhelmed (*n* = 1), and concern (*n* = 3). These results were reported across all articles, with some highlighting concern for the child’s health [36], while others drew conclusions based on parental mental health consultations (outpatients or ED) [37], without specifying the purpose of the consultation or differences between parents receiving positive versus negative results. The findings also indicated a moderate and short-term effect of stress [37–41]. Only one article [21] noted potential bias, as the most distressed parents may have declined to participate. Two articles highlighted the role of autonomy and desire for control in alleviating parental stress [42, 43]. Only one study examined parents at risk of depression, considering confounding factors [41], and found that stress associated with gNBS was transient and attributed by parents to other life stressors.

##### Significant impact may be more related to specific cases

Qualitative studies highlighted psychosocial consequences and the risk of false positives as key disadvantages, despite these not being primary barriers in quantitative research. Parents reported lacking adequate information, guidance, and psychosocial support during diagnosis, which caused stress [44]. While one study showed that mothers generally did not experience clinically significant anxiety, a subset reported decisional regret [45], suggesting that psychological impact may vary individually but still warrants assessment. Parental perceptions of condition severity after NBS, even without actual disease risk, contributes to parental stress, as seen in cystic fibrosis [46]. Concerns about false positives also led to unnecessary treatment and reduced quality of life [47], with mothers in false-positive groups reporting increased anxiety and concerns about their child’s development [48]. Long-term negative effects on parents following false positives have been reported [49], with parents of preterm infants showing more outpatient visits after false positives [50]. False positives significantly affect parental stress and the parent–child relationship, particularly when adequate information is lacking. Many parents expressed confusion about the purpose for retesting, with nearly half of those receiving false-positive results reporting that no clear explanation was provided for subsequent examinations [48]. The specific context and situation may carry a stress burden that is not entirely caused by gNBS itself.

##### Heightened stress for late onset diseases

Another specific concern arises when gNBS is used for late-onset conditions, as it can place a heavy psychosocial burden on families. Crossen et al. (2022) [51] reported that while parents recognised the benefits of early Pompe disease diagnosis, they also experienced grief and anxiety over the potential development and onset of symptoms. Diagnosed asymptomatic patients who do not require immediate treatment are referred to as “waitlist” patients, as they must live with the uncertainty of if or when the disease will appear, leading to anticipatory anxiety [51]. Uncertainty about symptom onset and treatment initiation can contribute to the medicalisation of children, seen in increased doctor visits, therapy, parental hypervigilance, and growing fear, anxiety, and uncertainty about the child’s future [52].

##### Fear also comes from fear of stigma

A second set of findings, connected to Parts 1 and 2, highlights concerns in the ethical literature about the potential for harm and/or stigmatisation of PLWRDs (*n* = 12). A major issue is the limitation of what is often referred to as the “open future,” affecting children and families labelled with conditions such as cystic fibrosis, resulting in psychosocial and emotional distress, compounded by the risk of ethnic discrimination. The ethical dimension of these programmes is critical, encompassing adequate risk communication, informed choice, and equitable access to balance expected benefits for some individuals against potential harms to others. Additionally, minimising false-positive screens and addressing differential sensitivities in ethnic groups for mutation panels are essential aspects of non-maleficence.

##### The role of support and communication in parental coping

Interviewed families reported difficult emotional reactions requiring support [44], including frustration with the initial communication of results and access to information [53]. Improved education for healthcare professionals and attention to stress arising from positive results for late-onset conditions may help [51]. Stress often stems from the uncertainty of vague results, necessitating comprehensive follow-up and communication [54]. Adequate communication and follow-up helped parents with inconclusive cystic fibrosis (CF) results understand information and perceive their child’s health as normal [33, 55]. Communication practices require improvement [56], highlighting the importance of comprehensive communication and follow-up [57]. Variability in the communication of positive NBS results is linked to resource limitations and a lack of guidelines [58], with systemic issues hindering the fulfilment of informational needs [50]. Ethical debates surrounding uncertainty and result management leave parents unsure about timelines. Managing secondary findings and cascade testing raises concerns about access and potential psychological harm from frequent follow-ups, creating “patients in waiting” for late-onset disorders. However, some parents felt that the non-medical benefits of gNBS, such as the knowledge it provides, outweighed concerns about the lack of available treatment for the screened disease.

#### Education is key for those who provide and those who receive it

From Part 1, we identified the need for improved education, encompassing the delivery, means, timing, and quality of information. Among the 34 articles retrieved, themed under Theme 1 from “Attitudes” (*n* = 13) or Theme 3 from “Barriers” related to educational content (*n* = 21), 13 articles addressed both themes [19, 21, 43, 46, 59–67]. Some research focused on the media—how education is provided to healthcare professionals, parents, or the public, including the internet, written information, and video content. Other studies examined communication strategies and behaviour, including the manner in which information should be delivered, effective wording (avoiding jargon), timing (when people wish to be informed), and the impact of bundled information on preferences. The source of information, typically healthcare professionals, was also considered, along with educational recommendations such as professional training, checking prior knowledge before delivering results, and guidance on managing information related to carrier status).

##### Communication of positive results: to whom and how ?

Information needs focused more on positive results than false positives. Communicating positive gNBS results to parents is challenging for healthcare professionals, who often lack formal support [68]. Positive results for asymptomatic infants are stressful and require clear communication to alleviate distress [54, 69]. Healthcare professionals should tailor disclosure to build trust [69]. Most parents prefer receiving initial positive gNBS results from knowledgeable healthcare professionals, ideally with both parents present [70–73]. Genetic counselling should address the impact of gNBS results on parenting. Sharing results outside the family varies; some parents find it helpful, while others find it upsetting [72]. Providers should adapt guidance to individual coping mechanisms [51, 53]. Emphasising informational and emotional support underscores the need for disease-specific education and strong parent–healthcare team connections, taking into account diverse cultural and educational backgrounds. Information should be individualised, practical, timely, efficient, and relevant to parents [20]. Several challenging aspects of gNBS diagnosis for parents require improvement [56].

##### Challenges for HCPs’ emotional ease

In Part 2s analysis of communication and information needs (*n* = 3), studies reported that healthcare professionals highly value their role, but only 62% feel comfortable with counselling. More than 80% of paediatricians and nurses reported having up-to-date knowledge, yet 40.4% considered their knowledge insufficient, and 55% requested further training, particularly for complex diseases [32]. Approximately 10% of paediatricians and 33% of family physicians reported feeling unprepared to discuss positive gNBS results for specific conditions [74]. Conversely, 54.5% of parents of infants with positive CF results reported wanting more information, and 13.8% suggested that primary healthcare professionals should be better informed about the details and implications of positive results [53].

Despite parental perceptions, many HCPs report confidence in their knowledge. For example, in one paper, 44% of family physicians reported that their knowledge is up-to-date [75], while a minority (16.5%) felt confident about sharing positive gNBS results [76]. They still reported obstacles to successful communication: insufficient time (42.2%), lack of compensation (52.2%), and lack of training (72.3%). Hayeems et al. (2021) [75] reported a change in HCPs’ perceptions regarding the importance of their role and their confidence in communicating positive screening results before and after reporting results. Initially, most rated their role as particularly important (88% of family physicians, 84% of paediatricians, and 98% of nurses), but confidence decreased after reporting positive results (64% of family physicians, 75% of paediatricians, and 80% of nurses), dropping to 54% among primary care providers reflecting on their experience in notifying parents of positive gNBS results. This decline can be explained by the discrepancy between expectations and actual outcomes.

##### HCPs welcome updating and training

Although HCPs have knowledge of gNBS, they require updated training to increase confidence and improve communication with parents. Key challenges include logistical issues, difficulty liaising with families and other HCPs, language barriers, parental reactions [68], time constraints, and limited access to genetics experts, particularly in rural areas [77]. Strong links between screening and diagnostic laboratories and primary care, along with ongoing education for primary care providers, are essential for improving the communication of positive gNBS results to parents [75].

Communicating positive gNBS results presents HCPs with diagnostic, prognostic, and therapeutic challenges, with uncertainty being a primary shared concern. Insufficient information increases parental uncertainty and emotional stress, and the way results are communicated can either ease or heighten these feelings [74]. Examining parents’ questions when receiving results, Chudleigh et al. (2022) [78] reported that a successful approach involves balancing precautionary care with avoiding over-medicalisation. Timely attention to this challenge is particularly important given the growing trend to include WGS in screening programmes [79].

#### “Actionability” potential is crucial for positive perception

##### Diverse terminology but unified intent to feel reactive

In most quantitative articles, the potential for actionability was clearly identified as an opportunity in some cases (theme 1), as it contributes to reducing the diagnostic odyssey and can also be a factor in barriers to gNBS (*n* = 5). Furthermore, it influences attitudes regarding gNBS (*n* = 12). Overall, 15 of 36 quantitative articles highlighted actionability factors. Articles presenting a “lack of actionability” as a barrier were cohort studies [19, 59, 80–83], reflecting the perspectives of one HCP study and four parent studies. When viewed in context, all 11 articles [43, 59–62, 64, 81, 82, 84–86] presenting actionability as the basis for a positive attitude were parent cohort studies, and some studies that noted actionability as a potential barrier ultimately coded it as having more benefits [81, 82]. Although all these articles considered actionability, only three explicitly used the terms “actionable” or “actionability” [21, 62, 64].

Qualitative research highlights the importance of actionability and treatability in gNBS decisions. Early diagnosis and treatment are the most frequently cited benefits. Parents are willing to pursue gNBS even if treatability is based solely on risky experimental treatments [51]. Studies show strong support for screening treatable disorders but less consensus for untreatable or late-onset conditions, suggesting optional screening in these cases [47, 87]. Parents prefer screening for severe or early-onset untreatable disorders [87] and believe they should have a choice in such instances. Some pregnant participants saw potential benefits in knowing about late-onset diseases for prevention through childhood interventions [88]. Parents weigh the negatives of foreknowledge of terminal illness against the benefits of social and financial preparation, support, and anticipating needs. Less common benefits include informing reproductive decisions, research participation, and joining support groups [87].

##### Extension of willingness to act in case of “carrier status”

Qualitative findings (*n* = 9) highlighted “carrier status” as a confusing concept for parents in the context of gNBS, with communication often debated and criticised. Articles reported a lack of information among carrier parents, emphasising the need for comprehensive communication and counselling to reduce parental stress, which involves diverse emotions during follow-up [57, 72]. The psychological challenges posed by conflicting carrier status messages require clear policies that consider the complexities of disclosure [89, 90]. Disclosure can be stressful due to the risk of discrimination and stigma, as carrier status, though medically benign, can carry social consequences [90]. Despite reported anxiety and confusion, most respondents with prior gNBS experience did not support withholding information about carrier status from parents [68].

##### The interplay with novelties in gNBS (extended gNBS, whole genome sequencing)

The connection between gNBS and actionable outcomes becomes less clear when whole-genome sequencing (WGS) is included, and parental attitudes toward WGS in gNBS remain limited and inconsistent [88]. Surveys indicate parental support for gNBS expansion and a desire for training and involvement [87], with WGS seen as increasing disease detection [87]. However, fewer parents were willing to participate in gNBS with WGS (80%) compared to conventional gNBS (94%) [91], raising concerns about off-target use [91]. Despite optimism about genomic gNBS, parents express concerns about privacy, control, discrimination, consent [88], ambiguous results [54], and family stress [92]. Health departments must ensure universal access while improving programmes that incorporate genomics [93]. Expanded gNBS with WGS presents challenges for paediatricians, who must manage a broader range of genetic diseases and require training for screening coordination and family communication [88]. The adequacy of extended gNBS is debated, with some paediatricians favouring mandatory inclusion [32], while others hold differing views on condition inclusion and data management [94]. There is no clear consensus among providers on WGS integration, including which conditions to screen, consent procedures, and result reporting [94].

#### Ethics and clinical utility

From the ethical papers, as well as from quantitative and qualitative results, specific ethical concerns regarding the clinical utility of screenings in general emerged. Treatability and actionability were central issues (*n* = 14). Critics argued that Wilson and Jungner’s criteria are too narrow, focusing solely on disease treatment. Additionally, targeted panels require constant updating with new genes, even for diseases that may never manifest clinically. Concerns about clinical utility are compounded by persistent uncertainties, such as VUS, uncertain phenotypes, and carrier status, which can result in unclear or inconsistent phenotypes. Nevertheless, the same literature highlights the benefits of NBS/gNBS, as it helps caregivers (even for non-treatable disorders) and HCPs prepare for and manage potential complications, expressed as ‘knowledge is power’.

##### Societal concerns mainly raised by HCPs and ethicists

In qualitative papers, gNBS was reported by HCPs as a potential burden for individuals and society. Ethical papers also emphasised its potential to strain the healthcare system, both structurally and economically (*n* = 5). Expanding NBS panels and using WES/WGS would increase incidental findings. For instance, emerging heterozygote risk data necessitates re-education of HCPs on genetic implications to identify at-risk infants, expanding care responsibilities and prompting system-level adaptations. Despite evidence supporting screening benefits, limitations in diagnostic capacity, treatment availability, and cost-effectiveness – particularly in low-prevalence contexts – underscore the need for tailored integration of gNBS into healthcare systems.

At the societal level, screening for specific RDs raises questions about equity and fairness in the gNBS process (*n* = 5), as access to a confirmed diagnosis varies depending on socio-economic status or national protocols in different countries. Moreover, even with advances in pharmacogenetics, the large volume of data raises concerns about potential biases in interpreting the ‘normal’ genome, since genetic testing often references specific genetic phenotypes. As some RDs are more prevalent in certain communities, gNBS could stigmatise minorities, given that mutation panels may have varying sensitivities across ethnic groups. Equity issues also extend to the care level after testing; if medical treatment is required, research indicates that limitations in genetic therapies and the restricted time available in primary care remain significant challenges.

##### Well “informed” consent still needed

Ethical papers questioned the adequacy of “informed consent,” as parents may not receive sufficient information to be truly informed before consenting (*n* = 9). This debate is linked to the presence – or absence – of an “opting out” option, since testing can be mandatory for some conditions. As the child cannot decide whether they wish to be tested, this can be considered as a violation of the “right not to know”, and ethicists have highlighted the potential risk of harm from obtaining predictive genomic information that children should be protected from. Although the importance of early identification may outweigh other social and ethical objections to gNBS, concerns remain regarding the limitations of informed consent in the context of expanded gNBS [92].

Consent issues also raise the question of who should be the decision-making agent: the child or the parents (*n* = 12). While some papers advocate for children’s assent to genetic testing, the newborn period precludes any possibility of such assent. Parents’ right to consent to screening for late-onset conditions raises concerns about respecting the child’s future autonomy, potentially resulting in parental choices that children may later contest. Even though guidelines emphasise respecting the child’s developing autonomy and prioritising the “right to an open future”, testing children without autonomous decision-making remains ethically debated. Result disclosure presents additional challenges for HCPs in addressing these ethical tensions, particularly regarding incidental findings and limited genetic counselling capacity. Although concerns are often minimised, unresolved questions remain about full disclosure, withholding heterozygous or borderline results, and guiding parental decision-making amid ethical tensions around information transparency.

##### Ethical challenges in gNBS arising from rare disease characteristics

The focus on specific RDs introduces additional ethical issues within gNBS. Privacy was a concern (*n* = 8), particularly regarding the organisation and communication of results. The literature highlights the risk of privacy breaches and debates over individual rights, with some arguing that parental access to a child’s genetic information may compromise confidentiality, as parents will have knowledge of the child’s results.

Another important ethical topic concerns the pre-symptomatic genetic testing of children for incurable diseases or late-onset disorders, which is considered ethically problematic due to unclear and limited direct benefits to the child and potential associated harms for specific diseases. The psychosocial impact of creating “patients-in-waiting” groups is closely linked to how rare diseases are selected for screening. These groups may include asymptomatic individuals who might never develop symptoms, potentially causing unnecessary parental stress [95].

#### Differentiating impacts: focusing on one rare disease or many

##### Disease-specific results

The selected articles emphasised the importance of distinguishing general “RDs” from specific RDs. Some qualitative studies focused on specific RDs – cystic fibrosis (CF), sickle cell trait or disease (SCT/SCD), fragile X syndrome (FRAXA), Pompe disease (PD), Krabbe disease, spinal muscular atrophy (SMA), and severe combined immunodeficiency (SCID) – when exploring WGS. Some research questions the inclusion of SCID and ataxia based on traditional gNBS criteria [4, 52, 96]. While positive results for CF and SCD have negative psychological impacts [33, 55], these could be mitigated with adequate information and genetic counsellor support [33]. Despite distress during Krabbe diagnosis, parents found screening useful and desired greater integration of genetic counselling [97]. For CF, initial education after diagnosis strongly influences parental adjustment and relationships with the care team [75]. Public support for gNBS for SMA was 84%, compared to 70% among families with affected children [92]. Parents of children with FRAXA, PD, and ataxia strongly support adding these conditions to gNBS for benefits such as immediate treatment [98, 99]. Most ataxia parents (81.1%) reported they would participate in gNBS if a test were available [52].

##### When considering all RDs

Parents reporting attitudes about RDs in general, primarily in qualitative studies (*n* = 25), emphasised the importance of pre-disclosure knowledge, disclosure consultations, and emotional support for gNBS. They want more information about diseases, especially those with rare symptoms, and repeated counselling throughout adolescence [73]. Understanding parental needs helps create tailored resources for families of children with RDs [71]. Effective information about positive outcomes for all RDs is crucial to reduce stigma [72]. Strategies are needed to expand counselling and improve genetic literacy regarding incidental results and carrier status related to RDs in gNBS [100]. Parental attitudes towards reporting carrier status vary by disease knowledge [89], but all agree on the need for professional counselling and timely medical information from HCPs. General HCPs reported difficulties counselling parents of children diagnosed with RDs, citing a lack of knowledge and experience [101]. Paediatricians showed knowledge gaps regarding sickle cell disease and CF, with only junior doctors reporting formal education on the medical or reproductive implications of these conditions [102]. Approximately 40% of primary healthcare providers lack knowledge to provide counselling about FRAXA [103], and 64% of GPs reported insufficient experience with SCD [101]. This highlights the need for improved paediatric genetic education for all healthcare providers to enable effective consent and counselling for families of children diagnosed with RDs [35, 103].

##### The ethical aspects of adjusting education to specificities

Additional prerequisites for improving diagnostic approaches include assessing differences in education, behaviour, ethnicity, and geographic location as social determinants. Living in a rural area, having lower education, and belonging to a minority may have an additive effect, potentially leading to worse outcomes for children diagnosed with RDs and their families [44]. A study on the social consequences of SCD in Africa examined the role of gender disparities before and after gNBS for SCD, assessing their impact on the quality of care for diagnosed children and recommending gender-specific health education and genetic counselling to improve community understanding of genetic diseases while reducing gender inequalities [104]. Bukini et al. (2021) reported that gender influences childcare and that discrimination affects both the quality of care and the mother’s identity, as she may be blamed for the child’s illness. Some family members may even distance themselves from the family, while the healthcare system can exacerbate gender imbalances by engaging mothers rather than fathers during follow-up, assuming they are more capable or willing to participate [104].

## Discussion

Our results show wide variation in sample sizes and a notable predominance of female participants. Differences between preference and epidemiology studies reflect distinct aims, effect sizes, and statistical requirements: preference studies often use smaller samples [105], whereas epidemiology studies require larger samples to detect smaller effects and ensure sufficient power [106]. Age ranges were highly variable, with adults grouped broadly from 15 to 91 years. As stated and actual attitudes may differ, sampling should closely reflect real-world situations to ensure accuracy [107]. Defining “reproductive age” is complex, varying from 15 to 44 years [108], 18–40 years in some contexts [109], or narrower ranges in specialised studies (e.g. 19–29, 30–39, 40–49) [110].

Demographically, results showed an over-representation of high-income countries, with only Jordan and China classified as middle-income (World Bank, October 13th, 2023). Although genetic training is expanding in LMICs alongside gNBS deployment [111–113], the literature still reflects a global focus on diagnosis and the expansion of specialised care for certain diseases, such as CF [114–116]. The overall goal of gNBS remains challenging, as efficient NBS programmes may still lack adequate follow-up or rehabilitation systems [117, 118], and public awareness of NBS for asymptomatic diseases remains limited [119]. Even in high-income countries, intra-national disparities persist, indicating that generalising gNBS (for both RDs and non-RDs) requires ongoing efforts to align screening programmes with effective care systems [120, 121]. Globally, gNBS priorities will differ, as education remains essential and infant mortality under five years remains a pressing issue [122]. Moreover, gNBS may risk widening existing inequalities, including racial ones [27, 123, 124].

Our results showed that ethical articles mainly originated from Western countries (USA, UK, Australia), with none from low- or middle-income countries. Historically, Western nations have shaped globally deployed ethical guidelines [125], and their worldwide generalisation may reflect a tendency to globalise perspectives that are difficult to apply locally or in non-Western contexts [126–128].

The most represented RDs were CF and sickle cell diseases, as they are the most established RDs [129, 130] and are included in almost all gNBS protocols worldwide [111]. Overall, the articles lacked consideration of prior experiences or the multifactorial nature of attitudes. Notably, no studies directly compared attitudes toward NBS and gNBS, limiting conclusions on the impact of the “genetic” dimension. This aligns with findings in other NBS research areas, such as hearing loss, where some parent samples (e.g. in Australia) showed low levels of depression, stress, and anxiety after positive results, without accounting for confounding factors reported in the literature [131]. This highlights the need to refine and validate research methods to ensure reliable evidence when studying small samples of parents facing multiple stressors in the postnatal period, in order to isolate the specific impact of a single variable (e.g., positive results) on their overall experience.

Future research could examine situations in which parents face uncertainty about a benign clinical course [132], compared with uncertainty linked to gNBS. Parents often struggle to form clear attitudes, and findings across NBS studies remain highly heterogeneous (see [133] for hearing loss [134]; for congenital hypothyroidism).

Another discussion point related to sampling and methods is the lack of a control group, as most articles did not compare parents receiving positive results with those receiving negative or false-positive results (except [21, 36, 86, 132, 135]). No study examined the attitudes of parents receiving false-negative results. As techniques advance, false-positive and false-negative results should decrease. In 2021, Kamleh et al. reduced false positives by nearly half (from 20.6% to 11.4%) using a TPN interruption protocol. Machine learning also shows promise; in hypothyroidism screening, it increased the positive predictive value by 21%, enhancing test discrimination. Despite disease-specific challenges, such advances could strengthen gNBS performance [136].

A notable point is that most qualitative studies did not focus on specific RDs, unlike quantitative studies. Future research could compare gNBS for individual RDs or “bundles” of RDs to assess whether the scope of information (broad versus specific) influences parental decision-making. Reporting non-treatable incidental findings remains controversial, as some policies recommend non-disclosure unless early detection can prevent significant harm to the child’s health [52]. This raises questions about how parents perceive benefits and harms in NBS decisions, considering disease treatability and test characteristics.

Our results show that ethical research generally precedes or coincides with qualitative and quantitative studies. This aligns with the evolution of empirical bioethics, which increasingly uses empirical data to inform policy and normative questions [137], incorporating psychosocial evidence into the “mapping–framing–shaping” approach to embed ethics within the research process [138]. Over the past decade, ELSI and bioethics have gained prominence in innovative research, aiming to generate behavioural and practical insights [139, 140], particularly in newborn screening, where ELSI questions are encouraged even at the pilot stage (see [141] for detailed research questions).

Our results indicate that most ethical papers were classified as “text and opinion”, consistent with literature showing a predominance of qualitative, descriptive quantitative, or mixed-methods studies in paediatric research on attitudes and understanding [142], and the frequent classification of ELSI papers as “text and opinion” when assessing their scientific rigour (e.g. SLR on ethical aspects of genetic testing for rare diseases) [143].

At the meta-theme level, our review aligns with current literature highlighting gNBS discussions on screening expansion with demonstrated feasibility [144] and the inclusion of novel techniques such as whole-genome sequencing and multi-omics [145], marking a new phase with ongoing debates and more precise questions about professional training and improved parental education. However, as in recent studies on expanded gNBS, parents generally remain interested in knowing their baby’s disease risks regardless of the method, showing limited variation in attitudes by NBS type [146]. Notably, mothers with greater genetic knowledge, higher education, or prior family genetic testing experience were more favourable toward genomic gNBS but still expressed concerns about the emotional and socio-ethical effects of genetic information on family and social dynamics [147]. Consistent with our findings, rigorous studies on the psychological impact of gNBS for RDs are lacking. Recent research indicates that receiving results for specific RDs can reduce parental stress over time, with differences between fathers and mothers, underscoring the need for more targeted studies on parental responses to gNBS for RDs [148].

With questions of new scalability, this raises the need for ethical consideration regarding the sustainability of gNBS for RDs in resource-poor countries [149], which were not addressed in the gNBS literature we reviewed. Fortunately, in recent years, the debate about actionability has become more open, as several initiatives have provided expanded genetic screening for actionable conditions [144], paving the way for potential acceptance of expanded gNBS [150]. Our results have several limitations. Our search did not focus on psychological measures or actionability/treatability, therefore conclusions on these topics are not systematic. The wide range of sample sizes may limit generalisability, and the predominance of women in study populations is a significant limitation. Relevant articles may have been missed due to unclear terminology, as “genetic newborn screening” is not consistently used compared with “newborn screening”. Additionally, not focusing on specific rare diseases may have excluded relevant findings for individual conditions (e.g. Pompe [151], FRAXA [152], SMA treatments [153], diagnostic accuracy [154], and broader childhood screening [155]).

The qualitative studies in our review were limited by sample size and composition, often involving few family members, which increases heterogeneity and reduces reliability and statistical power, though they provide diverse perspectives and analytical insights. Most studies relied on self-selected participants, potentially limiting population representativeness [92]. Short follow-up periods make it difficult to observe longitudinal dynamics. Our study focused on general attitudes rather than specific diseases and excluded cost-effectiveness research, which addresses socioeconomic aspects of genetic testing and could inform future studies on equitable healthcare access.

Our analysis of ethical aspects from an unrestricted search should not be considered a systematic review of ethics in gNBS for RDs. Some recent publications on gNBS for RDs [143] provide complementary insights, addressing ethical issues in non-newborn genetic screening. We did not limit “ethical aspects” to a defined set of principles (e.g. respect for autonomy, non-maleficence, beneficence, and justice, as in [143, 156]). Carrier status was not specifically excluded, though it raises distinct ethical concerns related to reproductive and adult decisions [157]. Finally, our exclusion of socio-economic factors limits consideration of policy, fairness, and equitable access to care [96, 158].

## Conclusion

In conclusion, the quality of gNBS research depends on careful selection of screened conditions and management of confounding factors such as prematurity and maternal history. Addressing Western-centric biases requires inclusive stakeholder engagement and broader global and ethnic representation. Clear terminology distinguishing key concepts (e.g. “treatability” versus “actionability”) and methodologies (gNBS versus traditional NBS) is critical. While attitudes toward gNBS are generally positive, our analysis highlights nuanced perspectives shaped by specific factors. Fully realising the potential of gNBS will require overcoming barriers in education, communication, and the complexity of genetic information, while addressing ethical issues of actionability, equity, and potential stigmatisation, especially for rare diseases and underrepresented populations [159].

## Electronic supplementary material

Below is the link to the electronic supplementary material.

Supplementary Material 1

Supplementary Material 2

Supplementary Material 3

Supplementary Material 4

Supplementary Material 5

## Data Availability

Data will be made available upon reasonable request to the corresponding authors and in accordance with Screen4Care data security procedures.

## Footnotes

1 The definition of actionability here refers to the “(a) conditions where early interventions lead to health gains for the newborn, (b) conditions where early diagnosis avoids the lengthy diagnostic delay and (c) conditions where parents will have reproductive options during subsequent pregnancies” (31Lombardo, S., et al., Policy-making and implementation for newborn bloodspot screening in Europe: a comparison between EURORDIS principles and UK practice *The Lancet Regional Health–Europe*, 2023. **33**, [32]. Ibid.

## Abbreviations

gNBS: genetic newborn screening,
NBS: newborn screening,
WGS: whole genome sequencing,
RD: rare disease,
HCP: healthcare providers,
DMD: Duchenne muscular dystrophy,
CF: Cystic Fibrosis,
FRAXA: Fragile X syndrome,
PD: Pompe disease,
MCAD: medium-chain acyl-coenzyme A dehydrogenase,
SCT/SCD: sickle cell traits or disease,
SMA: spinal muscular atrophy,
SCID: severe combined immunodeficiency,
VUS: variant of uncertain significance,
SLR: systematic literature review,
ELSI: ethical, legal and social implications,
UU: Uppsala University,
PRISMA: Preferred Reporting Items for Systematic Reviews and Meta-Analyses,
RCT: randomised controlled trial,
QoL: quality of life,
PLWRD: person living with rare diseases,
LMIC: low- and medium-income country,
WES: whole exome sequencing.
