# Supplementary material for: Preferences, attitudes and views regarding genetic newborn screening (gNBS) for rare diseases: a systematic review of the literature and synthesis from 2009 to 2022
Source: Orphanet J Rare Dis. 2026 Jan 8;21:27. doi: 10.1186/s13023-025-04179-0 (PMC12836846; doi:10.1186/s13023-025-04179-0)
Supplement: Supplementary file 1 — Supplementary Material 1 [file 13023_2025_4179_MOESM1_ESM.docx]

| PsycInfo (EBSCO), 2022-01-13, 2022-11-07 | | | |
| --- | --- | --- | --- |
| Search no | Search terms | Results | Comments |
| 1 | babies OR baby OR infant* OR neonat* OR newborn* [Title/Abstract] | 113,964 | 2022-01-13 |
| 2 | ENS OR "genetic test*" OR "genome test*" OR "genomic test*" OR "metabolic test*" OR NBS OR NGS OR "next generation sequencing" OR screen* OR "whole exome sequencing" OR "whole genome sequencing" OR WES OR WGS [Title/Abstract] | 112,265 | 2022-01-13 |
| 3 | Anxiety OR Attitudes OR Emotions OR Mental Health OR Public Opinion OR Stress [DE] | 337,383 | 2022-01-13 |
| 4 | angst OR anxiety OR attitude* OR emotion* OR expectation* OR experience* OR feedback OR feeling* OR mental OR opinion* OR perception* OR perspective* OR "point of view*" OR preference* OR psychological impact* OR stress* [Title/Abstract] | 2,209,194 | 2022-01-13 |
| 5 | #3 OR #4 | 2,239,529 | 2022-01-13 |
| 6 | Family OR Nurses OR Parents OR Physicians OR Siblings [MeSH] | 184,758 | 2022-01-13 |
| 7 | brother* OR clinician* OR counsellor* OR counselor OR dad* OR doctor* OR famil* OR father* OR gynecologist* OR "healthcare profession" OR kinship OR midwi* OR mother* OR mum* OR nurse* OR parent* OR personnel* OR physician* OR psychologist* OR relative* OR sibling* OR sister* OR staff* OR therapist* [Title/Abstract] | 1,388,363 | 2022-01-13 |
| 8 | #6 OR #7 | 1,396,686 | 2022-01-13 |
| 9 | #1 AND #2 AND #5 AND #8  Publication date 2005 🡪 | 1,178 | 2022-01-13 |
| 10 | Publication date 2022/01/13 🡪 | 81 | Update 2022-11-07 |

| PubMed, 2022-01-12, 2022-11-07 | | | |
| --- | --- | --- | --- |
| Search no | Search terms | Results | Comments |
| 1 | Infant, Newborn [MeSH] | 644,449 | 2022-01-12 |
| 2 | babies OR baby OR infant* OR neonat* OR newborn* [Title/Abstract] | 803,220 | 2022-01-12 |
| 3 | #1 OR #2 | 1,104,106 | 2022-01-12 |
| 4 | Genetic Testing OR Neonatal Screening OR Whole Exome Sequencing OR Whole Genome Sequencing [MeSH] | 73,989 | 2022-01-12 |
| 5 | ENS OR "genetic test*" OR "genome test*" OR "genomic test*" OR "metabolic test*" OR NBS OR NGS OR "next generation sequencing" OR screen* OR "whole exome sequencing" OR "whole genome sequencing" OR WES OR WGS [Title/Abstract] | 961,815 | 2022-01-12 |
| 6 | #4 OR #5 | 994,507 | 2022-01-12 |
| 7 | Anxiety OR Attitude OR Emotions OR Mental Health OR Public Opinion OR Stress, Psychological [MeSH] | 1,093,985 | 2022-01-12 |
| 8 | angst OR anxiety OR attitude* OR emotion* OR expectation* OR experience* OR feedback OR feeling* OR mental OR opinion* OR perception* OR perspective* OR "point of view*" OR preference* OR psychological impact* OR stress* [Title/Abstract] | 3,688,260 | 2022-01-12 |
| 9 | #7 OR #8 | 4,183,658 | 2022-01-12 |
| 10 | Family OR Nurses OR Parents OR Personnel, Hospital OR Physicians OR Siblings [MeSH] | 667,864 | 2022-01-12 |
| 11 | brother* OR clinician* OR counsellor* OR counselor OR dad* OR doctor* OR famil* OR father* OR gynecologist* OR "healthcare profession" OR kinship OR midwi* OR mother* OR mum* OR nurse* OR parent* OR personnel* OR physician* OR psychologist* OR relative* OR sibling* OR sister* OR staff* OR therapist* [Title/Abstract] | 4,406,532 | 2022-01-12 |
| 12 | #10 OR #11 | 4,643,586 | 2022-01-12 |
| 13 | #3 AND #6 AND #9 AND #12  Publication date 2005 🡪 | 4,499 | 2022-01-12 |
| 14 | Publication date 2022/01/13 🡪 | 479 | Update 2022-11-07 |

| Scopus, 2022-01-13, 2022-11-07 | | | |
| --- | --- | --- | --- |
| Search no | Search terms | Results | Comments |
| 1 | babies OR baby OR infant* OR neonat* OR newborn* [Title/Abstract] | 950,771 | 2022-01-13 |
| 2 | ENS OR "genetic test*" OR "genome test*" OR "genomic test*" OR "metabolic test*" OR NBS OR NGS OR "next generation sequencing" OR screen* OR "whole exome sequencing" OR "whole genome sequencing" OR WES OR WGS [Title/Abstract] | 20,822,660 | 2022-01-13 |
| 3 | angst OR anxiety OR attitude* OR emotion* OR expectation* OR experience* OR feedback OR feeling* OR mental OR opinion* OR perception* OR perspective* OR "point of view*" OR preference* OR psychological impact* OR stress* [Title/Abstract] | 946,361 | 2022-01-13 |
| 4 | brother* OR clinician* OR counsellor* OR counselor OR dad* OR doctor* OR famil* OR father* OR gynecologist* OR "healthcare profession" OR kinship OR midwi* OR mother* OR mum* OR nurse* OR parent* OR personnel* OR physician* OR psychologist* OR relative* OR sibling* OR sister* OR staff* OR therapist* [Title/Abstract] | 8,485,517 | 2022-01-13 |
| 5 | #1 AND #2 AND #3 AND #4  Publication date 2005 🡪 | 3,455 | 2022-01-13 |
| 6 | Publication year 2022 | 794 | Update 2022-11-07 |

| Web of Science, 2022-01-13, 2022-11-07 | | | |
| --- | --- | --- | --- |
| Search no | Search terms | Results | Comments |
| 1 | babies OR baby OR infant* OR neonat* OR newborn* [Title/Abstract] | 721,204 | 2022-01-13 |
| 2 | ENS OR "genetic test*" OR "genome test*" OR "genomic test*" OR "metabolic test*" OR NBS OR NGS OR "next generation sequencing" OR screen* OR "whole exome sequencing" OR "whole genome sequencing" OR WES OR WGS [Title/Abstract] | 1,136,512 | 2022-01-13 |
| 3 | angst OR anxiety OR attitude* OR emotion* OR expectation* OR experience* OR feedback OR feeling* OR mental OR opinion* OR perception* OR perspective* OR "point of view*" OR preference* OR psychological impact* OR stress* [Title/Abstract] | 6,797,938 | 2022-01-13 |
| 4 | brother* OR clinician* OR counsellor* OR counselor OR dad* OR doctor* OR famil* OR father* OR gynecologist* OR "healthcare profession" OR kinship OR midwi* OR mother* OR mum* OR nurse* OR parent* OR personnel* OR physician* OR psychologist* OR relative* OR sibling* OR sister* OR staff* OR therapist* [Title/Abstract] | 6,170,072 | 2022-01-13 |
| 5 | #1 AND #2 AND #3 AND #4  Publication date 2005 🡪 | 2,623 | 2022-01-13 |
| 6 | Publication year 2022 | 428 | Update 2022-11-07 |
