# Supplementary material for: Preferences, attitudes and views regarding genetic newborn screening (gNBS) for rare diseases: a systematic review of the literature and synthesis from 2009 to 2022
Source: Orphanet J Rare Dis. 2026 Jan 8;21:27. doi: 10.1186/s13023-025-04179-0 (PMC12836846; doi:10.1186/s13023-025-04179-0)
Supplement: Supplementary file 2 — Supplementary Material 2 [file 13023_2025_4179_MOESM2_ESM.docx]

**Supplementary material 2: Psychological scales:**

The structured scales were used assessing different pathological and non-pathological elements in quantitative article : the Edinburgh Depression Scale (N=2), State-Trait Anxiety Inventory (N=4), Impact of Event Scale ( n=2), Vulnerable Baby Scale (n=2), Parenting Stress Index (PSI) - Short Form (n=3), Kansas Marital Satisfaction Scale, Depression measure, Generalized Anxiety Disorder Scale, Patient Health Questionnaire, Edinburgh Postnatal Depression Scale (EPDS)(n=2), Beck Anxiety Inventory,  Hospital Anxiety and Depression Scale, Center for Epidemiological Studies Depression Scale.

Other scales were also used in the qualitative papers : the 36-item short form of the Parenting Stress Index; the Spielberger State-Trait Anxiety Inventory; the Edinburgh Postnatal Depression Scale; the Quality of Life Inventory (QOLI); and the Decision Regret Scale. The Emotional Intimacy Subscale of the Personal Assessment of Intimate Relationships Inventory was used to assess spousal support.
