# Supplementary material for: Preferences, attitudes and views regarding genetic newborn screening (gNBS) for rare diseases: a systematic review of the literature and synthesis from 2009 to 2022
Source: Orphanet J Rare Dis. 2026 Jan 8;21:27. doi: 10.1186/s13023-025-04179-0 (PMC12836846; doi:10.1186/s13023-025-04179-0)
Supplement: Supplementary file 3 — Supplementary Material 3 [file 13023_2025_4179_MOESM3_ESM.docx]

**Supplementary material 3**: Inclusion and exclusion criteria

| **Description** | **Inclusion criteria** |
| --- | --- |
| **Population** | Parents, mothers, fathers, HCPs (incl. clinicians, midwives, nurses, psychiatrists, etc.), decision makers/ politicians |
| **Testing** | Newborn Screening (NBS), including genetic NBS (gNBS) |
| **Comparators** | Not restricted by comparators |
| **Outcomes** | Parents' perception of gNBS for metabolic disease |
|  | Parents' experience with gNBS |
|  | Parents' acceptance of gNBS |
|  | HCPs' perception of gNBS |
|  | HCPs' experience with gNBS |
|  | HCPs' acceptance of gNBS |
|  | Ethical aspects in gNBS |
|  | Post-gNBS experiences and feedbacks |
| **Study design** | Any study design |
| **Language restrictions** | English only |
| **Publication type** | Any publication type except books, chapter, conference abstract, "conference review", editorial, erratum, letter, note, "review", short survey or tombstone |
| **Country restrictions** | Not restricted by country, but focus on experience that can relatable to European country inhabitants |
| **Date restrictions** | between 2009 and 2022 |

Final selection criteria were written in English language between 2009 to 2022, data collection no earlier than 2009, exclusion of articles focused on some of the following conditions - hypothyroidism, congenital deafness (hearing screening), heart defects (pulse oximetry) or NICU-sick babies gNBS for the clinical aspects (for more details about type of publication see Table 1). At the final selection we decided to consider an extended definition of *“genetic newborn screening”* therefore including studies about NBS using genetic procedure or delivery of RDs NBS results for newborns that experts identified as part of gNBS even if the exact term gNBS was not used(Institute, 2023, 17th November) as an exclusion/inclusion criteria but if NBS was considered as a comparison to gNBS, the article was still included. The included articles covered various facets of the "genetic" aspect within the papers, including a focus on genetic or rare disease diagnosis, expanding NBS to include genetic or rare diseases information, and addressing false-positive concerns in complex NBS scenarios that were not focused primarily on gNBS but that were identified as relevant for the present study by a blind assessment of 3 reviewers to reach consensus.
