# Supplementary material for: Preferences, attitudes and views regarding genetic newborn screening (gNBS) for rare diseases: a systematic review of the literature and synthesis from 2009 to 2022
Source: Orphanet J Rare Dis. 2026 Jan 8;21:27. doi: 10.1186/s13023-025-04179-0 (PMC12836846; doi:10.1186/s13023-025-04179-0)
Supplement: Supplementary file 4 — Supplementary Material 4 [file 13023_2025_4179_MOESM4_ESM.docx]

Annexe Part 1: Included articles description

| **First Author et al.** | **Title** | **Year** | **Study/ Research Endpoint/-s AIMS** | **Population Type** 1. General Public 2. HCPs 3. Patients 4. Parents  5. Relatives/ Caregivers 6. Other/ Mixed 7. N/A | **Sample size (n)** **number of participants who successfully completed the study* | **Gender ratio** (% male, % female) | **Age range** (if available) | **Country/-es** | **Overall Conclusion -  "Take-Home Message"** (1 or 2-sentence summary) |
| --- | --- | --- | --- | --- | --- | --- | --- | --- | --- |
| Lillie et al. (1) * | Framing optional genetic testing in the context of mandatory newborn screening tests | 2015 | Assess if the presentation of a case in isolation or alongside the total number of mandatory NBS test changes the intention to participate in DMD NBS. | 1. General Public | 2991 | 54% male, 46% female | 21–82 | USA | Participants were more likely to choose optional DMD NBS if they also read information about mandatory NBS (either bundled or unbundled) versus when DMD NBS was presented in isolation. |
| Temme et al.(2) | Assessment of Parental Understanding of Positive Newborn Screening Results and Carrier Status for Cystic Fibrosis with the use of a Short Educational Video * | 2015 | The aim of this study was to improve parental understanding of positive NBS results using an educational video in addition to genetic counseling. | 3. Parents | 96 | 41.7% male,58.3 % female | N/A | USA | Videos in addition to written materials aid in the understanding of results and information about specific conditions. |
| Christie et al.(3) | Maternal Attitudes to Newborn Screening for Fragile X Syndrome* | 2013 | The aims of this study were (1) to test both male and female newborns for FXS to determine feasibility and accuracy of two concurrent testing methodologies; (2) to determine postnatal mothers’ acceptance and attitudes to screening and reasons for accepting or declining participation; and (3) to assess the impact of diagnosis of a child with an abnormal result. | 4.PArents | n=1971 (parents), n=2000 patients | 100% female mother | 21-40 | Australia | Mothers found NBS for FXS acceptable, and valued an early diagnosis because of the opportunity to prepare for a child with additional needs. There are common reasons for non-participation: a perceived low risk of having a child with FXS, not wanting to know, declining all NBS, concerns about carrier testing in children, a preference to wait for symptoms to occur before testing, the waiting time for results being too long, and that there is no cure for FXS. |
| Cyrus et al. (4) | Clinic based infant screening for Duchenne Muscular Dystrophy: A feasibility study* | 2012 | Questioning parents of infants regarding their experience of screening of DMD. | 6, Children and Parents | 266 | N/A | N/A | USA | Approximately 78% of parents indicated support of voluntary DMD screening and 91% of providers were in favor of screening for DMD. Parents in general understood benefits and risks, later screening allows additional time for parent-child bonding. |
| Davids et al. (5) | Health care practitioners' experience-based opinions on providing care after a positive newborn screen for Pompe disease* | 2021 | This study explores HCPs experiences and challenges providing NBS patient care throughout the US and their resultant opinions on NBS for Pompe Disease (PD). | 2.HCPs | 78 | N/A | N/A | USA | While NBS for PD is viewed positively by HCPs serving this population, there are challenges that must be addressed to maximize outcomes (medical, psychological, and financial), particularly for individuals with LOPD. |
| Evans et al. (6) | Assessing the newborn screening education needs of families living in medically underserved areas* | 2020 | Asses the NBS education needs of families living in medically underserved areas (MUA) | 4. Parents | 500 in total, 200 MUA | 34,6% male, 65,4% female | N/A | USA | NBS online education program is desirable to families across geographic areas. Families living in the USA have less access to NBS information and have special need for communication strategies. |
| Farrell (7) | Frequency of high-quality communication behaviors used by primary care providers of heterozygous infants after newborn screening* | 2013 | Assess the impact of communication quality indicator data (quality of communication, jargon use / clarification, precautionary empathy, assessment of understanding, organizing behaviors) | 2,HCPs | 214 | 41.1% male; 58.9% female | N/A | USA | The limited use of high-quality communication behaviors in rehearsals raises concern about parental understanding, decision-making, and psychosocial outcomes after newborn screening. |
| Farrell et al. (8) | Vulnerable Child Syndrome and Newborn Screening Carrier Results for Cystic Fibrosis or Sickle Cell * | 2020 | Recall Parental perceptions of child vulnerability assessed by adapted version of the Vulnerable Baby Scale | 6.426 parents of SCH carriers, 288 parents of CF carriers and 79 in the clinic comparison group | 426 parents of SCH carriers, 288 parents of CF carriers and 79 in the clinic comparison group | 98% mothers of SCH carriers, 96,5% of mothers of CF carriers | mean 25,8 y for SCH carrier group, 28,7 for CF carrier group,30 for clinic comparison group | USA | Increased parental perceptions of child vulnerability and risk for Vulnerable Child Syndrome are bona fide risks of incidental findings after NBS identifies carrier status. Population-scale mechanism for follow-up after carrier identification to ensure "more good than harm". |
| Farrell et al.(9) | Experience with Parent Follow-Up for Communication Outcomes after Newborn Screening Identifies Carrier Status* | 2020 | Assess what were the variable in communication based on 5 elements ((1) whether the parent recalled being told about the NBS result, (2) whether the parent recalled the provider giving an explanation, (3) parent's appraisal of the explanation, (4) parental satisfaction with the entire experience, and (5) misconception about risk for carrier status developing into the actual disease.) | 3. Parents | 426 parents of SCH carriers, 288 parents of CF carriers | 98% mothers of SCH carriers, 96,5% of mothers of CF carriers | mean 25,8 y for SCH carrier group, 28,7 for CF carrier group | USA | With the NBS expansion more effort to mitigate harm should be invested. |
| Fitzgerald et al. (10) | Newborn bloodspot screening for cystic fibrosis: What do antenatal and postnatal women know about cystic fibrosis?* | 2016 | Knowledge studies about antenatal (≥36 weeks gestation) and postnatal women (post NBS) about CF knowledge | 4.Parents | 1142 | 100% female (mothers) | 32.2 years (± 5.12) | Ireland | Women that participated in this study appear to have good knowledge about CF; however, there were some misconceptions about CF . Different demographic and maternity characteristics are related with the level of CF knowledge. |
| Genetti et al. (11) | Parental interest in genomic sequencing of newborns: enrollment experience from the BabySeq project* | 2019 | Assessing the reasons for enrollment refusal in pre and post ES | 4.PArents | 3860 &1760 declining participations | N/A | 33.6 | USA | Reason to decline before Enrollment was burdensome logistic(48%), feeling overwhelmed with postpartum (17%), lack of interest (17%) Decliner after enrollment session had concerns about privacy/insurability (41%) and uncertain/unfavorable results (23%). still lots of concerns explain declining participation. |
| Hayeems et al. (12) | False-Positive Newborn Screening for Cystic Fibrosis and Health Care Use * | 2017 | Using cystic fibrosis (CF) as an example, determine the association of FP NBS results with health care use in infants and their mothers | 6.Newborns and mothers | FP, N = 1564 ; Screen-Negative, N = 6256 |  | Maternal age 30 (26–34) | Canada | Higher use of outpatient services among FP infants may relate to a lengthy confirmatory testing process or follow-up carrier testing. However, increased rates of hospitalization might signal heightened perceptions of vulnerability among healthy infants. |
| Karaceper et al. (13) | The health system impact of false positive newborn screening results for medium-chain acyl-CoA dehydrogenase deficiency: a cohort study * | 2016 | Comparing false positive NBS for MCADD compared with negative NBS, and also matched controls regarding health care utilisation (physician visits, ER visits and hospitalisations) | 3.Patients | 43 false positive children; comparison cohort 545,355 children;  matched comparison cohort 420 children | 65% male 35% female | 0-6 years | Canada | Higher use of some health services among false positive infants during the first year of life. This may be explained by a psychosocial impact of false positive results on parental perceptions of infant health, and/or by differences in underlying health status. |
| Kasem et al. (14) | Mothers’ knowledge and attitudes about newborn screening in Jordan* | 2022 | Examine the knowledge and attitudes of mothers regarding the newborn screening test in Jordan | 4.PArents | 301 | 100% female | 18-40 | Jordan | The mothers’ source of information about the test was a significant predictor for both their level of knowledge and attitudes toward the newborn screening. The healthcare providers, particularly nurses, were identified as the main source of mothers’ information in Jordan. |
| Nardini et al. (15) | Genomic Counseling in the Newborn Period: Experiences and Views of Genetic Counselors* | 2014 | on-line survey regarding experience with counseling for NBS and WGS, preparedness to counsel for sequencing results from NBS, including current disorders on panels, WES results, and WGS results. | 2.HCPs | 208 | 3.4% male (n=7) 96,6% female (n=201) |  | USA | Counselors expressed ethical and practical concerns regarding WGS in NBS, as well as a need for additional training regarding this application of the technology before it is implemented (genetic counselor do not always feel comfortable counselling on WGS for NBS). |
| Paquin et al. (16) | Parental intentions to enroll children in a voluntary expanded newborn screening program* | 2016 | Examine parental intentions to participate in voluntary expanded screening. | 4.Parents | 1001 | 21.3% male 78.7 females | 18- 45+ | USA | Program design features may impact the psychological mechanisms underlying parental decision making for voluntary expanded screening. |
| Sadat et al. (17) | Increased parental anxiety and a benign clinical course: Infants identified with short-chain acyl-CoA dehydrogenase deficiency and isobutyryl-CoA dehydrogenase deficiency through newborn screening in Georgia* | 2020 | Looked at the clinical outcomes of children with SCADD or IBDD as well as assessment of the parental anxiety alongside it. | 6.General public and patients | 60 children and 18 parents | for children with SACDD 48% female 52% male for children with IBDD 50% male and 50% female for parents it was not captured | mean for children only 5-6 years of age | USA | Both NBS programs and clinical genetics practices should consider how they handle SCADD and IBDD, and also consider developing a coordinated strategy that takes into account the impact of all changes throughout the system. |
| Tluczek et al.(18) | Psychosocial Consequences of False-Positive Newborn Screens for Cystic Fibrosis* | 2011 | To develop a more comprehensive picture of parents’ perspectives of NBS by examining parents’ understanding of their infants’ false-positive NBS results and how their knowledge of an infant’s carrier status affected them and their interactions with other family members during the infant’s first year of life. | 4.PArents | 87 | Not captured | mean age 29 years for mothers; mean age 30 years for fathers; | USA | Abnormal NBS results that involve genetic testing can have psychosocial repercussions that affect entire families. These findings merit additional investigation of long-term psychosocial sequelae for false-positive results, interventions to reduce adverse iatrogenic outcomes, and the relevance of the relational family system framework to other genetic testing programs. |
| Vernooij-van Langen et al. (19) | Parental knowledge reduces long term anxiety induced by false-positive test results after newborn screening for cystic fibrosis* | 2014 | Assess disease knowledge test score and HADS (hospital anxiety and depression score) | 4.Parents | 62 parents from the FP group and 146 parents from the control group | 21.2% male; 78.8% female | 19-43 years | The Netherlands | False-positive results in NBSCF lead to strong negative feelings immediately after the positive test, but do not seem to cause long-term parental anxiety. Well-informed parents showed less anxiety and depression, which shows the importance of providing parents and professionals with adequate educational material. |
| Wade & Elliott (20) | Young adults’ attitudes toward pediatric whole-genome sequencing* | 2016 | aims to characterize the views of young adults on the provision of pediatric WGS, the communication of WGS findings during childhood and what it would have been like to grow up knowing one’s WGS results. | 1.General public | 145 | 37% male; 63% female | 18-19 years | USA | It will be important to integrate the perspectives of children and young adults. The prospect of children growing up with knowledge of their genetic risks presents a range of ethical and policy challenges that should be addressed prior to widespread implementation, in addition to crucial research that clarifies the clinical utility of pediatric WGS. |
| Waisbren et al. (21) | Psychosocial Factors Influencing Parental Interest in Genomic Sequencing of Newborns * | 2015 | Assessment of Parental Stress Index, health concerns encountered in the first months of the babies' life, and psychological factors | 4.Parents | 663 | 32.9% male; 67.1% female | 15-65 years | USA | Results suggest that parental stress and worrisome prenatal/perinatal experiences will influence parental interest in GS when it is offered. Additional research on factors motivating parents to accept or reject GS will clarify how and when parents should be approached. |
| Lang et al. (22) | Maternal Attitudes About Sickle Cell Trait Identification in Themselves and Their Infants* | 2010 | Examine maternal knowledge and attitudes about SCT and SCD in women whose infants were identified with SCT (FAS) through NBS to help determine lay public knowledge and attitudes and where educational programs should be focused | 4. Parents | 100 | 100% female | average age 26 years | USA | Health care professionals need to improve the timely reporting and communication about prenatal and neonatal SCT results to women who are frequently unaware that they or their infants had hemoglobinopathy testing. When informed, most women are willing to disclose their child’s SCT to first-degree relatives and their child’s pediatrician, although how the recipients use and understand this information has not been adequately explored. |
| Weinreich et al. (23) | Public support for neonatal screening for Pompe disease, a broad-phenotype condition* | 2012 | Assess support of a (neutral) consumer panel and (parents of) patients with Pompe disease in 2010. | 6. neutral group; parents; patients | 555 neutral; 58 Pompe-experienced informants | neutral 38% male, 62 % female; Pompe 33%male, 67% female; | neutral 21-91 years Pompe 31-74 years | The Netherlands | This survey suggests a rather high level of support for newborn screening for Pompe disease, not only among those who have personal experience of the disease but also among the general public in the Netherlands. Optional screening on the basis of informed parental consent is probably unrealistic, underlining the need for new guidelines to help policymakers in their consideration of newborn screening for broad phenotype conditions. |
| Wood et al. (24) | Parental attitudes toward newborn screening for Duchenne/Becker muscular dystrophy and spinal muscular atrophy* | 2014 | Assess the attitudes toward NBS for DMD, BMD, and SMA were surveyed and compared for 2 categories of parents, those with children affected with DMD, BMD, or SMA and expectant parents unselected for known family medical history | 4.Parents | MDA Cohort: 72 MUSC Cohort: 22 Magee Cohort: 408 | MDA Cohort: 25% male; 75% female MUSC Cohort: 27.3% male; 72.7% female Magee Cohort: 42.0 % male; 52.7% female | MDA Cohort: 20-40+ MUSC Cohort: 20-40+ Magee Cohort: <20->50 | USA | Both parents of affected children and expectant parents find NBS for DMD, BMD, and SMA acceptable despite the current lack of curative treatment. Given advances in diagnostics and promising therapeutic approaches, discussion of inclusion in NBS should continue. |
| Wright et al. (25) | Understanding Midwives’ Preferences for Providing Information About Newborn Bloodspot Screening* | 2018 | Collect a preference survey including a conjoint analysis (CA) and discrete choice experiment to quantify preferences for the types of, and way in which, information should be provided in a perspective of an expanded newborn bloodspot screening program. | 2.HCPs | 134 | 0.8% male; 99.2% female | 16-65+ years | UK | A wide variety of information should be provided to maximize parents’ ability to make a decision ideally provided late in pregnancy or on day 3 post birth. |
| Wright et al. (26) | Eliciting Preferences for Information Provision in Newborn Bloodspot Screening Programs* | 2017 | A hybrid choice experiment, combining a conjoint analysis and a discrete choice experiment in the context of expansion of NBS established national programs | 4.PArents | 702 | 42% male; 58% female | 18-45 years | UK | Parents’ preferences for receiving NBS information differ from how this information is given in current UK practice. Future work is needed to understand the financial and economic impact of new models of information provision in the context of a UK-based NBSP, particularly when there is a continued call for expansion of screening programs to include more conditions. |
| Farrell et al. (27) | Parental Preferences about Policy Options Regarding Disclosure of Incidental Genetic Findings in Newborn Screening: Using Videos and the Internet to Educate and Obtain Input | 2022 | To develop and test a new approach to obtaining parental policy guidance about disclosure of incidental findings of newborn screening for cystic fibrosis (CF), including heterozygote carrier status and the conditions known as CFTR-related metabolic syndrome (CRMS) and/or cystic fibrosis screen positive inconclusive diagnosis, CFSPID | 4.Parents | 128 respondents | 81.3% women (mothers) 18,7% men (fathers) | median respondent age was 33 | USA | Most parents strongly preferred the policies of full disclosure or making the decision, rather than the withholding option for NBS results. Data from value comparisons suggested that parents believed knowing everything was very important even if they became distressed. Likewise, parents preferred autonomy even if they became distressed. |
| Ong et al. (28) | Knowledge, Attitudes, and Awareness Towards Newborn Screening in Association with Received Prenatal Care: A Survey of Primiparous Postpartum Mothers at the Philippine General Hospital * | 2022 | check how and if mother get views on NBS. knowledge, attitude, and awareness of primiparous postpartum mothers towards NBS | 4.Parents | 160 | only mothers | <20 to 40. 20-29 years old (56.3%) <20 years old (35.6%) 30-39 (7.5%) ± 40 (0.6%) | Philippines | There is no significant difference between the mean attitude score of those with prenatal checkup and those without. There is lack of consent or information to the parents about the use of results as a source for research whether they had previous prenatal consults or not. |
| Peay et al. (29) | Education and Consent for Population-Based DNA Screening: A Mixed-Methods Evaluation of the Early Check Newborn Screening Pilot Study* | 2022 | check for understandability and acceptability of population-based DNA screening | 4.Parents | 1824 survey 24 interviews | N/A | median age from 32 to 35 | USA | Data from programs that use virtual education and consent may lead to best practices in new material development and may increase the acceptance of participant-centered electronic consent among regulators. |
| van der pal et al. (30) | Parents’ views on accepting, declining, and expanding newborn bloodspot screening* | 2022 | Assess the reason for non-participation in GNBS and attitudes in Dutch gNBS program | 4.Parents | 804 | 657 women | mean age 32.2 | The Netherlands | Parents agree to prevent health complaints, whereas the most important reason to decline NBS was parents’ viewpoint on life and the belief that the heel prick would be painful for the child. The one rejecting NBS were more actively religious, considered alternative medicine or lifestyle more important, were less inclined to vaccinate their child for infectious diseases, and reported more doubt about NBS participation. Rejection was associated with significant lower/insufficient knowledge |
| Wang et al. (31) | Current attitudes and preconceptions on newborn genetic screening in the Chinese reproductive‑aged population | 2022 | surveying attitudes at maternity hospital of Nanjing | 4.Parents | 1141 | female ration 1:4 | mean age 31 (± 4) years | China | Most individuals were willing to accept NBGS programs. Those with a high level of education and income provide a solid foundation for the future development of NBGS. The cost of NBGS testing, on the other hand, may hinder the development of NBGS in countries with low-income and lower literacy rates. Yet, the development of NBGS may be reasonably simple and easy for high-income countries and for educated people who understand its importance for the family in the longer term |
| Wu et al. (32) | Are We Ready for Newborn Genetic Screening? A Cross-Sectional Survey of Healthcare Professionals in Southeast China | 2022 | To understand the knowledge, attitude, willingness, and ability of healthcare professionals working in newborn screening (NBS) centers regarding newborn genetic screening (nGS) | 2.HCPs | 258 | Male 43 (16.67%) Female 215 (83.33%) | <18 to >50 | China | Most participants were interested and considered nGS necessary. The inability to provide genetic counseling may be the primary impediment to clinical practice. Three important influencing factors were level of education, institution level, and engagement in TMS screening. |
| Plass et al. (33) | Neonatal Screening for Treatable and Untreatable Disorders: Prospective Parents’ Opinions * | 2009 | Opinion of prospective parents concerning newborn screening for disorders that are incurable yet treatable to some extent or even untreatable. | 4.Parents | 1631 | 96.5% female 3.5% male | not captured | The Netherlands | In contrast to current policy, respondents showed a positive attitude toward inclusion of less treatable (88%) or untreatable childhood-onset disorders (73%) within the national newborn screening program. |
| Pereira et al. (34) | Psychosocial Effect of Newborn Genomic Sequencing on Families in the BabySeq Project A Randomized Clinical Trial* | 2021 | Surveys with parents of newborns as well as baby nurseries and ICUs about psychological effects | 6.Parents and Newborns | 325 | 47% male 53 % females parents | not captured | USA | Parents and clinicians are less confident in GS than NBS, but parents perceive a more favorable risk/benefit ratio of GS than do clinicians. Clinicians should be aware that parents’ optimism may stem from their perceived benefits beyond clinical utility. Parents find their children’s genomic information useful for a broad variety of reasons, including psychological and pragmatic reasons, which traditionally fall outside of the realm of what is considered clinical utility. |
| Oyeku et al. (35) | Primary Care Clinicians’ Knowledge and Confidence About Newborn Screening for Sickle Cell Disease: Randomized Assessment of Educational Strategies * | 2010 | RCT Assessment of pre and post educational interventions/surveys with Clinicians | 2.HCPs | 49 | 20% male 80% female | not captured | USA | Both educational strategies led to modest improvements in knowledge about newborn screening for hemoglobin disorders. Enhancing knowledge and confidence about newborn screening-related tasks may improve clinicians’ capacity to act upon newborn screening results for hemoglobinopathies. Psychosocial consequences of a false positive newborn screening test are short-term for the majority of parents, studies suggest that a small proportion continue to experience anxiety following diagnostic testing. |
| Schwartz et al. (36) | Effects of participation in a U.S. trial of newborn genomic sequencing on parents at risk for depression* | 2021 | Aimed to determine if parents’ depressive symptoms were associated with their child's participation in a randomized-controlled trial of newborn exome sequencing. | 4.PArents | 82 | N/A | N/A | N/A | Participation in a randomized-controlled study of ES in newborns, which included the return of results in the sequencing arm did not raise significant concerns for parents. |
| Carlton et al. (37) | Acceptability of childhood screening: a systematic narrative review* | 2021 | to assess via a SLR the acceptability of childhood screening interventions to identify factors to consider when planning or modifying childhood screening programs to maximize participation and uptake | N/A | N/A | N/A | N/A | N/A | The main acceptability component identified related to parental knowledge and under-standing of the screening process, the testing procedure(s), and consent. The emotional impact of childhood screening mostly explored maternal anxiety. |
| Tluczek et al. (38) | Psychosocial Issues Related to Newborn Screening: A Systematic Review and Synthesis* | 2022 | Address Psychosocial consequences of NBS, parents’ knowledge of and attitudes towards NBS, reactions to and understanding of positive NBS results, experiences of communication with health providers, decisions about carrier testing, and future pregnancies | 4.PArents | N/A | N/A | N/A | N/A | Psychosocial consequences of receiving unexpected neonatal screening results and unsolicited genetic information remain significant risks to expansion of NBS. Findings suggest that risks may be mitigated by improved parent NBS education, effective communication, individualized genetic counseling, and anticipatory developmental guidance. Clinicians need to take extra measures to ensure equitable service delivery to marginalized subpopulations. |
| Clark & Boardman (39) | Expanding the notion of “benefit”: comparing public, parent, and professional attitudes towards whole genome sequencing in newborns* | 2022 | comparing attitudes among parent general public and HCPs | 6. HCPs, general public, parents | N/A | N/A | N/A | UK | The general public and parents supported the use of WGS-NBS in principle, as they perceive wide-ranging benefits to their child, family and society – even if those benefits were not immediately realizable. This panoramic view of benefit neutralized many of the risks they identified. |

Annexe Part 2:

| **First Author et al.** | **Title** | **Year** | **Study/ Research Endpoint/-s AIMS (if described)** *objective measure or parameters being evaluated; example "looking at biomarkers", "enzyme activity" "genetic variant", etc.* | **Population Type** 1. General Public 2. HCPs 3. Patients 4. Parents  5. Relatives/ Caregivers 6. Other/ Mixed 7. N/A | **Population size (n)** **number of participants who successfully completed the study* | **Gender ratio** (% male, % female) | **Age range** (if available) | **Country/-es** | **Overall Conclusion -  "Take-Home Message"** (1 or 2-sentence summary) |
| --- | --- | --- | --- | --- | --- | --- | --- | --- | --- |
| Acharya et al. (40) | A Pilot Study to explore Knowledge, Attitudes and Beliefs about Sickle Cell Trait and Disease* | 2009 | Examine the knowledge, attitudes, beliefs and disclosure patterns about SCT of parents | 4.Parents | 53 adults | N/A | N/A | USA | Significant misinformation about what it means to be a carrier and its health and reproductive implications |
| Acharya & Ross (41) | Fragile X Screening: Views of Genetic Health Professionals* | 2009 | Assess attitudes about FrX testing and population screening from Survey of physician geneticists and genetic counselors. | 2.HCPs | 273/894 | 28% male, 72% female | N/A | USA | GHP prefer preconception screening and screening to identify boys and girls with both pre- and full mutations |
| Acharya & Schindler (42) | Developmental and Behavioral Pediatricians Attitudes Toward Screening for Fragile *X | 2013 | Assess attitudes of DBP towards FMR1 newborn screening and FMR1 carrier testing from Survey of Developmental and Behavioral Pediatricians. | 2.HCPs | 294/484 | 36% male, 63% female | N/A | USA | DPB support FMR1 NBS, DBP are not familiar with the array of FXAD. |
| Araia et al. (43) | Factors associated with knowledge of and satisfaction with newborn screening education: a survey of mothers* | 2012 | Survey of mothers about knowledge and satisfaction with NBS education, Assess knowledge and satisfaction of mothers with NBS education | 4.PArents | 712 | 100% female | 91% of participants were >25 years of age | Canada | Promoting mothers’ understanding and meeting their expectations with respect to education about newborn blood-spot screening may require greater engagement with prenatal providers. |
| Pruniski et al. (44) | Newborn screening for Pompe disease: impact on families* | 2018 | Looking at impact of NBS for Pompe disease on families | 6. Parents and Patients | 11 | 100 % female | Not captured | USA | LOPD families are coping with pre-symptomatic genetic knowledge (aka patients-in-waiting).Increased fear/anxiety and living with uncertainty (regarding diagnosis, their children’s future, and when to start treatment) were predominant themes, with all families voicing considerable emotional reactions and varied social and healthcare support concerns |
| Bailey et al. (45) | Caregiver Opinions about Fragile X Population Screening* | 2012 | Assess caregiver opinions about fragile x population screening via Survey of caregivers about fragile x population screening. | 6.Parents and caregivers | 1099 | 87% were mothers | 16-89 | USA | Caregivers of children with Fragile X widely endorse screening. |
| Boardman et al. (46) | Newborn genetic screening for spinal muscular atrophy in the UK: the views of the general population | 2017 | attitude towards NBS for SMA (even if no type I, II or III) based on SMA patients for survey and general population, | 6.Patients and family members | 36 interviews and survey of 337 adults and families | 74.4% | from 35 to 55 years (52%) | UK | This study demonstrates that newborn screening for SMA is viewed largely positively by people unfamiliar with the condition. The importance of early identification overrode all other social and ethical concerns about screening for the majority of participants |
| Boardman(47) | Attitudes toward population screening among people living with fragile X syndrome in the UK:'I wouldn't wish him away, I'd just wish his frigile X syndrome away'* | 2020 | interview with families and little number of patients of FX about screening views, interviews with (family of) people with fragile X syndrome about different screening options (NBS but also prenatal or preconception) | 6.parents, spouse, grandparents, sibling, aunt | 17 | 84% female | 17-55 | UK | Screening could be beneficial for diagnosis of fragile X syndrome |
| Bombard et al. (48) | Public views on participating in newborn screening using genome sequencing* | 2014 | Assess willingness to participate, parental responsibility to participate and science attitude towards : Whole genome sequencing vs NBS | 1.General public | 1213 | 48% female | 18- >55 | Canada | Integrating WG/ES into NBS might reduce participation, and challenge the moral authority that NBS programmes rely upon to ensure population benefits. These findings point to the need for caution in the untargeted use of WG/ES in public health contexts. |
| Bukini et al. (49) | Perspectives on Building Sustainable Newborn Screening Programs for Sickle Cell Disease: Experience from Tanzania * | 2021 | In-depth interviews and focus group discussions. 3 main themes, Pre-Screening, Post-Screening and Sustainability strategies | 6.Families, HCP, Policy Makers | n=40 total, n=4 policy makers, n=15 families, n=7 HCPs (no nurses), n=14 nurses | N/A | N/A | Tanzania | Pre-Screening, Post-Screening and Sustainability strategies proved acceptability and accessibility of NBS, NBS for SCD programs in Tanzania have demonstrated both the opportunities and areas that need addressing in the implementation and sustainability of the services in low resource settings. |
| Burlina & Corsello (50) | Survey of Italian pediatricians’ perspectives and knowledge about neonatal screening* | 2015 | To assess their familiarity and opinions on newborn screening in general and on expanded newborn screening. | 2.HCPs | n=605 | N/A | N/A | Italy | Pediatricians may need additional training to allow them to fulfill their tasks of coordinating NBS. |
| Cao et al. (51) | Australian healthcare professionals' perspectives on the ethical and practical issues associated with genomic newborn screening* | 2022 | Assess Australian HCPs' perspectives regarding GNBS - the perspectives of healthcare professionals (HCPs) in Australia on the ethical and practical issues associated with the implementation of genomic newborn screening (GNBS) - HCPs perspectives on the ethical, social, and practical issues raised by integrating GS into NBS | 2.HCPs | 16 | n-a | n-a | Australia | There was a lack of clear consensus among HCPs such as how GS may be integrated, what type of conditions to include, which group of HCPs is most suitable to obtain informed consent, and what type of results should be returned following GNBS. |
| Chudleigh et al. (52) | Qualitative exploration of health professionals’ experiences of communicating positive newborn bloodspot screening results for nine conditions in England | 2020 | To explore health professionals' experiences of communicating positive newborn bloodspot screening (NBS) for rare diseases results, highlight differences, share good practice and make recommendations for future research | 2.HCPs | 17 | N/A | N/A | UK | Great variation in the way positive NBS results are communicated to parents and this is largely influenced by resources available but also the lack of concrete guidance. HPs invest a lot of time and energy trying to make sure communication of positive NBS results to families is done well. |
| Chudleigh et al. (53) | Parents’ Experiences of Receiving the Initial Positive Newborn Screening (NBS) Result for Cystic Fibrosis and Sickle Cell Disease* | 2016 | Semi-structured, qualitative interviews to explore parents' experiences of receiving the initial positive NBS result for their child with cystic fibrosis (CF) or sickle cell disease (SCD). | 4.PArents | n=12 mother, n=10 fathers | N/A | N/A | UK | Genetic counselling needs to include a focus on the impact of NBS results on parental relationships. Effective communication and SCD management is vital to help reduce perceived stigmatisation associated with having a child with SCD. |
| Conway et al.(54) | Pain points in parents’ interactions with newborn screening systems: a qualitative study* | 2022 | To investigate parents’ experiences navigating the NBS system, with a particular focus on their developing information needs over the course of their NBS journey and elucidate parents’ experience of newborn screening [NBS], with the overarching goal of identifying desiderata for the development of informatics-based educational and health management resources. | 6.General public and parents | 35 parents | 34 female | - 25 to 47 years | USA | A wide range of experiences of, and attitudes towards the newborn screening program and the wider newborn screening system. While parents’ view of the screening process was – on the whole – positive, some participants reported experiencing substantial frustration, particularly related to how results are initially communicated and difficulties in accessing reliable, timely information. |
| Crossen et al. (55) | A Qualitative Study: Mothers’ Experiences of Their Child’s Late-Onset Pompe Disease Diagnosis Following Newborn Screening* | 2022 | Recall mothers’ experiences of their child’s LOPD diagnosis and medical monitoring | 4.PArents | 8 | - 100% women | - aged between 29 and 38 years old; median - 35 | USA | Newborn screening for conditions with both early onset and late-onset forms, like Pompe disease, can result in challenges for families and providers. It is important to recognize the psychosocial burden of newborn screening for late onset disease. |
| De Luca et al. (56) | Parents' experiences of expanded newborn screening* | 2011 | Describe open-ended interviews before and/or after parents received confirmatory test results for their infants | 4.PArents | 30 families, in total 44 parents | 14 couples and 16 solo mothers | not captured | USA | The evaluation of a newborn for an abnormal screening result was highly stressful for parents. To help reduce parents’ distress, improvements in communications and clinical services are needed. Almost every parent in the study believed that newborn screening was an important program that preserved the health of infants through early identification and treatment of disorders |
| Jessup et al. (57) | Parental Experience of Information and Education Processes Following Diagnosis of Their Infant With Cystic Fibrosis Via Newborn Screening* | 2016 | semi-structured interview, to understand parents' experiences of initial education following their infant's diagnosis with CF following newborn screening (NBS) | 4.PArents | 10 | 7 women 3 men | 33-52 years | Australia | There is no ‘one-size-fits-all’ for information delivery. From an initial stance of impaired receptivity, effective education informs the individual, initiates them into the world of CF, helps them instigate a care regime, integrates them into the care team, and empowers them to achieve this extra-ordinary, unanticipated level of parenting. |
| Johnson et al. (58) | Psychological Impact on Parents of an Inconclusive Diagnosis Following Newborn Bloodspot Screening for Cystic Fibrosis: A Qualitative Study* | 2019 | Interpretative phenomenological analysis (IPA) on psychological impact of CFSPID designation following NBS for CF | 4.PArents | 8 | 5 women 3 men | N/A | UK | Findings suggest that CFSPID results caused parents’ distress, initiated with the first communication of the result and persisting thereafter. |
| Joseph et al. (59) | Parental Views on Expanded Newborn Screening Using Whole-Genome Sequencing * | 2016 | Focus groups with socioeconomically and ethnically diverse pregnant women and a comparison group with parents of children diagnosed with a primary immunodeficiency disorder to collect Parental Views on Expanded Newborn Screening Using Whole-Genome Sequencing | 4.PArents | 8 | 5 women 3 men | N/A | UK | Findings suggest that CFSPID results caused parents’ distress, initiated with the first communication of the result and persisting thereafter. |
| Kusyk et al. (60) | A Pilot Study to Evaluate Awareness of and Attitudes About Prenatal and Neonatal Genetic Testing in Postpartum African American Women | 2013 | Two side study with 1) to determine maternal SCT correlates with prematurity and/or low birth weight 2) Questionnaire surveyed the ethical, legal, and social implications (ELSI)of SCT. | 4.PArents | 60 | 100% female | Mean 26.4 ± 5.6 SD | USA | Despite professional guidelines that stress the importance of education, counseling, and consent for prenatal and neonatal testing, postpartum women do not recall these conversations |
| La Pean et al. (61) | A qualitative secondary evaluation of statewide follow-up interviews for abnormal newborn screening results for cystic fibrosis and sickle cell hemoglobinopathy * | 2012 | To examine parents’ reactions to NBS follow-up in a richer and more descriptive fashion than is possible with more traditional, Likert-scaled evaluation surveys. | 4.PArents | 195 | 2.6% male, 97.4% female | <20-49 | USA | Parents of CF and SCH carrier infants had favorable opinions and identified specific benefits to receiving follow-up contact. This analysis demonstrates an information deficit among carrier parents and illustrates the importance of NBS follow-up and need for comprehensive communication and counseling. |
| Lang et al. (62) | Maternal Knowledge and Attitudes About Newborn Screening for Sickle Cell Disease and Cystic Fibrosis* | 2009 | To examine maternal attitudes and knowledge about NBS for SCD and CF, about the genetics, symptoms and treatments for SCD and CF, and how women learn about NBS. | 4.Parents | 388 | 100% female | 18–46 years | USA | Postpartum women report inadequate education about NBS, but nonetheless, they are supportive of it. Creative educational efforts are needed to promote health literacy in mothers of all educational backgrounds. |
| Leppert et al. (63) | Genetic Counselors’ Experience with and Opinions on the Management of Newborn Screening Incidental Carrier Findings* | 2018 | Evaluate genetic counselors’ attitudes about disclosure of carrier status results generated by NBS and to gather data on their experiences with incidental carrier findings | 2.HCPs | 235 | 4% male, 96% female | 20-70 | USA | The data suggest that implementation of an opt-in/out policy for parents to decide whether or not to receive incidental findings would be beneficial. The results of this study support the continued disclosure of incidental carrier findings; however, additional research is necessary to further determine and implement the most effective disclosure practices. |
| Lipstein et al. (64) | Parents’ Decision-Making in Newborn Screening: Opinions, Choices, and Information Needs | 2010 | Using framework analysis, analyze how parents’ preferences varied according to disease characteristics, test characteristics, and perceptions of the associated risks and benefits when making decision about gNBS | 4.PArents | 45 | 9% male, 91% female | N/A | USA | Parents’ preferences differed according to experience with genetic conditions. However, most parents desired more-detailed information and suggested some optional testing. |
| Lisi & McCandless (65) | Newborn Screening for Lysosomal Storage Disorders: Views of Genetic Healthcare Providers* | 2016 | To ascertain the opinions and experiences of genetic healthcare providers working with patients with inborn errors | 2.HCPs | 38 | Not captured | Not captured | USA, Canada | In general, HCPs’ opinions regarding criteria for screening are consistent with those elucidated by Wilson and Jungner 50 years ago: diseases included on the NBS should have effective treatment, younger age of onset, straightforward test results, and the natural history and prognosis should be well established. |
| Mak et al. (66) | The first pilot study of expanded newborn screening for inborn errors of metabolism and survey of related knowledge and opinions of health care professionals in Hong Kong* | 2018 | To investigate the feasibility of expended NBS for health care professionals by convenience sampling. | 6.parents HCPs | 1. 2 440 neonates (pilot study) 2. 210 HCPs (NBS survey) | Not captured | 1. 24 - 48 h (84,6% neonates), 3 - 5 days (13,6%), 5 - 7 days (0,4%), 7 - 28 days (1,5%) - pilot study 2. HCPs - not specified | China | Health care professionals support implementation of newborn screening for IEM; however, there is a substantial need of more education. |
| Noke & Ulph (67) | Young Adults’ Pre-Existing Knowledge of Cystic Fibrosis and Sickle Cell Diseases: Implications for Newborn Screening* | 2013 | To explore young adults’ pre-existing knowledge of CF and SCD and ability to assimilate disease information, with a view to understand how adults’ pre-existing knowledge profiles influence engagement in a NBS context. | 6. University students | 34 | 32.4% male 67.6% female | 18-26 | UK | Young adults’ prior disease knowledge should be considered within a newborn screening context and written materials should consider the inclusion of carrier statistics to improve information relevance. |
| Miller et al. (68) | Understanding sickle cell carrier status identified through newborn screening: a qualitative study* | 2010 | Assess stakeholders attitudes toward communicating results of carrier status for SCD | 6. 1. HCPs community advocates - SCD, parents of SCD-carrier infants, lay consumers | 1. 56 - interviews 2. 66 - focus groups | Interviews: 34% male (19) 66% female (37) Focus groups: 10,6% male (7) 89,4% female (59) | Not captured | Canada | Different groups have different positions on reporting carrier status for SCD, also depending on their knowledge. |
| Moody et al. (69) | Healthcare professionals’ and parents’ experiences of the confirmatory testing period: a qualitative study of the UK expanded newborn screening pilot * | 2017 | Analyze views and experiences of HCPs and parents on communication and interaction during the period of confirmatory testing following a positive NBS result | 6. Parents of children with positive ENBS result HCPs with experience on positive screening result | 1. 10 parents 2. 11 HCPs | - Parents: 3 male (30%) 7 female (70%) | Not captured | UK | A number of elements within the path through confirmatory testing that are difficult for parents and could be further developed to improve the experience. These include the way in which the results are communicated to parents, rapid turnaround of results, offering a consistent approach, exploring interventions to support family relationships and reviewing the workload and scheduling implications for healthcare professionals. |
| Nnodu et al. (70) | A Multi-centre Survey of Acceptability of Newborn Screening for Sickle Cell Disease in Nigeria* | 2018 | To ascertain the attitudes to and acceptability of NBS in Nigeria among various sociodemographic groups including health professionals, undergraduate students, parents of children with SCD and SCD patients. | 6. HCPs undergraduate students, parents of children with SCD and SCD patients. | 1301 | 51.6% male/46% female 2% did not indicate gender | 19-49 | Nigeria | This study suggests that there is a good acceptability of NBS across Nigeria. The main barriers to its use are likely to be financial and practical, rather than social or cultural. |
| Peterson et al. (71) | A qualitative assessment of parental experiences with false-positive newborn screening for Krabbe disease* | 2022 | Assess experiences of parents with false positive on Krabbe disease | 4.Parents | 12 | 10 mothers 2 males | na | USA | Need to improve understanding of the NBS process from a parent perspective / the role of healthcare provider communication / the value of Krabbe NBS. |
| Prakash et al. (72) | Newborn screening for Pompe disease: Parental experiences and follow-up care for a late-onset diagnosis* | 2022 | To assess the impact of LOPD identification through NBS, different need for follow-up. exploring the differences in attitudes, emotions and opinions among parents and identify their needs for follow-up care. | 4.Parents | 10 families | 9 mothers 1 father | na | USA | Predominantly, parents reported a lack of adequate information, guidance, and psychosocial support from the very beginning and through the course of their diagnosis. This caused uncertainty, anxiety, frustration, and fear of the unknown. Understanding parents' experiences allows genetic counselors and NBS programs to proactively design care plan for parents during this difficult period |
| Raspa et al. (73) | Information and Emotional Support Needs of Families Whose Infant Was Diagnosed With SCID Through Newborn Screening* | 2020 | To evaluate information needs among parents about newborn screening and the implications of having a positive diagnosis for a rare disorder. | 4.Parents | Parents (n = 76) | (13.16% male, 86,84% female) | N/A | USA | Findings from the parent needs assessment activities will serve as the foundation for creating a suite of resources for those who have a child with SCID. |
| Salm et al. (74) | Informing parents about positive newborn screen results: Parents’ recommendations* | 2012 | To address gaps in the empirical literature by obtaining parents’ perspectives about how best to communicate positive NBS results. | 4.Parents | 203 | (50,7% male, 49.3% female) | N/A | N/A | The quality of informants’ communications with families can have a powerful effect in exacerbating or alleviating parents’ negative reactions arising from receipt of positive NBS results. Sensitive and effective communication with parents about positive NBS results can have the added benefit of fostering mutually gratifying parent–provider relationships. |
| Perobelli et al. (75) | Inconclusive Cystic Fibrosis neonatal screening results: long-term psychosocial effects on parents* | 2009 | Determine the long-term psychosocial effects on parents of newborns with inconclusive Cystic Fibrosis NBS results. | 6.Parents and Newborns | 33 | not captured | not captured | Italy | Parents of children, where the CF neonatal screening procedure led to inconclusive results, and who were offered adequate communication and clinical follow-up, understood properly the information received, and had a correct perception of their children’s health status, which they considered not different from their peers’. |
| Sims et al. (76) | Parents’ Experiences and Needs Regarding Infant Sickle Cell Trait Results* | 2022 | Explore parents’ experiences with and desires for SCT disclosure and counseling for their infants with SCT identified via newborn screening | 4.Parents | 16 | 9 female | na | USA | Five themes were identified: parent knowledge before child’s SCT disclosure, family planning, the dynamics of SCT disclosure and counseling, emotions and actions after SCT disclosure, and parent desires for the SCT disclosure and counseling process. Parents want more information about SCT, particularly rare symptomatology, and they want SCT counseling repeated once the child approaches adolescence. |
| Stark et al. (77) | A Pilot Study to Evaluate Knowledge and Attitudes of Illinois Pediatricians toward Newborn Screening for Sickle Cell Disease and Cystic Fibrosis* | 2011 | Measure knowledge and attitude of pediatricians towards NBS. | 2.HCPs | 337 | (37% male, 63% female) | N/A | USA | Knowledge gaps among AAP-affiliated Illinois pediatric providers about SCD and CF. This highlights the need for greater genetic educational outreach for pediatricians. |
| Nicholls & Southern (78) | Parental Decision-Making and Acceptance of Newborn Bloodspot Screening: An Exploratory Study* | 2013 | To understand the factors that influence parental decisions and roles they play in the decision-making process. | 4.Parents | 18 | Not captured | not captured | UK | While content is important, other contextual factors such as personal experience, perceived choice, and general attitudes toward medicine, are also highly influential. In particular, relationships with key HCP are central to information collection, attitudes toward screening, and the level of deliberation that is invested in decisions to accept newborn bloodspot screening |
| Tarini et al. (79) | False-Positive Newborn Screening Result and Future Health Care Use in a State Medicaid Cohort* | 2011 | To conduct a population-based study by using state Medicaid claims to explore whether infants with false-positive NBS results had greater future healthcare use than infants with normal NBS results. | 3.Patients | 49 959 infants | (50,9% male,49.1 % female) | >37weeks and <37 weeks | USA | Medicaid-insured term infants with FP NBS results did not have more health care visits than those with normal NBS results. Preterm infants with FP NBS had more acute outpatient visits than those with normal NBS. |
| Timmins et al. (80) | Diverse Parental Perspectives of the Social and Educational Needs for Expanding Newborn Screening through Genomic Sequencing* | 2022 | Explore opinions of expanding NBS, ethical and privacy concerns, and educational and social needs. | 4.Parents | 35 | 100% female | 24-47 mean 33 | USA | Parents support the expansion of NBS through GS. Furthermore, they desire earlier education and greater participation in the process. |
| Tluczek et al. (81) | Factors Associated With Parental Perception of Child Vulnerability 12 Months After Abnormal Newborn Screening Results * | 2011 | To explore equation derived from: illness frequency, parental perception of child vulnerability (Child Vulnerability Scale) and Parenting Stress (Parenting Stress Index) | 4.Parents | 257 | 47.08% male, 52.92% female | mean age 30.9 years | USA | The research suggests that perceived seriousness of a condition identified through NBS is a critical factor in whether parents develop Parental Perception of Child Vulnerability and associated parenting stress. |
| Tu et al. (82) | Psychological Effects of False-Positive Results in Expanded Newborn Screening in China* | 2012 | To assess if Parental stress and parental-child relationship, as well as factors influencing these end-points such as education/income/knowledge of NBS are present | 4.Parents | 129 | 31.78% male, 68.22% female | mean age 29.7 years | China | False-positive screening results may affect parental stress. This is especially true for parents who have not received adequate information about newborn screening. Therefore, Parental stress and anxiety can be reduced with improved education and communication to parents about false positive results. |
| Ulph et al. (83) | Familial influences on antenatal and newborn haemoglobinopathy screening* | 2011 | To explore knowledge of universal antenatal and newborn screening for haemoglobin disorders, impact of family on screening decisions, sources of support, impact of screening results on service user (parent) and their family | 4.Parents | 37 | Not captured | mean age mothers 32 years, mean age fathers 34 years | UK | Providing sufficient resources to empower probands to inform family and friends and challenge stigmatising beliefs is likely to not only increase community knowledge, but also resources which aid adaptation to such information. |
| van Dijk et al. (84) | Expanding Neonatal Bloodspot Screening: A Multi-Stakeholder Perspective * | 2021 | Exploration on perspectives on (1) Benefits and challenges of the current expansion of NBS, (2) Future expansions: new developments and broadening the aim of NBS, and (3) NBS acceptance and consent procedures. | 6.Parents and screening professionals | 22 professionals; 17 parents | Not captured | 27-40 years | The Netherlands | The ongoing expansion of the Dutch NBS program seems to be supported by the different groups of stakeholders, especially as it follows the rationale of health gain for the newborn. In order to maximize support from professionals, parents and society for the program, it is important that stakeholders with different views at least find themselves heard. |
| Vansenne et al. (85) | Providing Genetic Risk Information to Parents of Newborns with Sickle Cell Trait: Role of the General Practitioner in Neonatal Screening* | 2011 | Aim as (1) to evaluate compliance of the GPs to the NBS program according to the instructions given by letter, (2) to assess the knowledge about hemoglobinopathies, and (3) to explore the potential risks and benefits of disclosing carriers of SCD reported by GPs. | 2.HCPs | 131 | 55% male; 45% female | 31-61 years | The Netherlands | We conclude by stating that the goal of reporting carrier status for hemoglobinopathies in universal NBS in the Netherlands has not been achieved in the observed period. Since GPs reported few barriers in counseling parents and only indicate a lack of knowledge and limited clinical experience, more efforts are needed to provide better information to GPs and to help facilitate their work. |
| Koopmans & Ross (86) | Identification and Management of Sickle Cell Trait by Young Physicians* | 2012 | To evaluate SCT education during paediatric residency, current NBS follow-up practice, and awareness of the NCAA policy | 2.HCPs | 355 | 30% male; 70 % female | Not captured | USA | Despite formal SCT education, a significant number of pediatricians do not verify NBS results or counsel about the medical implications of SCT. More comprehensive AAP guidelines about SCT are needed and must be incorporated into residency education. |
| Hayeems et al. (87) | Primary care role in expanded newborn screening* | 2013 | Address Beliefs, practices, and barriers related to providing information to families who receive positive screening results for their newborns. | 2.HCPs | 710 | 31.4% men 68.6% women | Not captured | Canada | In the context of caring for families of infants who receive positive screening results, this study endorses an information-provision role for primary care providers, efforts to mitigate barriers to pursuing this role, and more fulsome inquiry into defining the actual scope of this role |
| Hayeems et al. (88) | Primary care providers’ role in newborn screening result notification for cystic fibrosis * | 2021 | To explore primary care providers role in result notification following a NBS. | 2.HCPs | 321 | survey: 63.4% female 36.6% male interview: 91.2% female 8.8% male | N/A | Canada | Close links between screening and diagnostic laboratories and PCPs, as well as ongoing educational initiatives in primary care, will be essential for effective delivery of genomic medicine. |
| Goldenberg et al. (89) | Genomics and Newborn Screening: Perspectives of Public Health Programs* | 2022 | Assess the benefits and challenges of using genomics in Newborn Screening Programs (NBS) from the perspectives of State program officials; and to help programs develop policies that will aid in the integration of genomic technology | 6. State program officials - NBS Program and Laboratory Directors | over 100 participants | Not captured | Not captured | USA | Benefits of incorporating genomics included improving screening modalities, supporting diagnostic procedures, and screening for a wider spectrum of disorders. Challenges included the costs of genomics, the ability to educate parents and health care providers about results, and the potential negative psychosocial impact of genomic information. Attempts to address the challenges of integrating genomics must focus on preserving the child welfare goals of NBS programs. |
| Bukini et al. (90) | Influence of gender norms in relation to child’s quality of care: follow-up of families of children with SCD identified through NBS in Tanzania* | 2021 | Collecting views via in-depth interviews with families of children with SCD and focus group sessions with nurses working in neonatal and postnatal sections | 6.Mothers, Couples, Nurses | 15 families, 14 nurses | N/A | N/A | Tanzania | NBS and gender roles in Tanzania, women will be blamed and not the men, they will think SCD is from the mother’s side and may even decide to stay far away from the family and this is because of the paternalistic societies we live in. |
| Azzopardi et al. (91) | Health-care providers’ perspectives on uncertainty generated by variant forms of newborn screening targets * | 2020 | Assess perspectives of HCP about the approaches for uncertainty of NBS variants | 2.HCPs | 12 | 7 female, 5 male | N/A | Canada | HCP have concerns about challenges associated with uncertainty of NBS variants and also prefer that management strategies for variant forms of screening targets err on the side of caution. Transparent guidelines are needed. |
| Bailey et al. (92) | Maternal Consequences of the Detection of Fragile X Carriers in Newborn Screening* | 2015 | To determine whether premutation mothers experienced adverse mental health outcomes and regretted screening and how they adapted over time | 4.PArents | 15 | 100% female | 18-44 | USA | The premutation group was not statistically different from the comparison group on measures of anxiety, depression, stress, or quality of life. A subset of mothers experienced clinically significant anxiety and decision regret |
| Blom et al. (93) | Dilemma of Reporting incidental findings in newborn screening programs for SCID: parents perspective on ataxia telangiectasia* | 2019 | To look at pro and cons of early vs late testing of children when non treatable disease | 4.PArents | 659 | 86.9% female | 34,7 for mothers 32,1 for dads | The Netherlands | The majority of parents of healthy newborns are in favor of an early A-T diagnosis in the pre-symptomatic phase of the disease. Moreover, the majority of parents would use a screening test for A-T, if such a test were available. Decisive arguments to participate were the fact that early detection of A-T prevents a long period between the first symptoms and the diagnosis and that early detection of A-T ensures immediate optimal guidance for a child when the first symptoms occur. |

**Annexe part 3.**

Annexe Part 3:

| **First Author et al.** | **Title** | **Year** | **Study/ Research Endpoint/-s AIMS (if described)** | **Population Type** 1. General Public 2. HCPs 3. Patients 4. Parents  5. Relatives/ Caregivers 6. Other/ Mixed 7. N/A | **Population size (n)** **number of participants who successfully completed the study* | **Gender ratio** (% male, % female) | **Age range** (if available) | **Country/-es** | **Overall Conclusion -  "Take-Home Message"** (1 or 2-sentence summary) |
| --- | --- | --- | --- | --- | --- | --- | --- | --- | --- |
| Botkin (94) | Ethical issues in pediatric genetic testing and screening for current opinion in pediatrics* | 2017 | Assess how progress in gNBS technology also brings ethical concerns in pediatrics, stemming from the often limited clinical usefulness of genetic data, the emergence of secondary findings, and uncertainties regarding parental testing authority. | 7. N/A | N/A | N/A | N/A | N/A | Adverse psychosocial impacts from genetic testing and screening in children. The use of these technologies is expanding with the notion of personal utility of test results is considered sufficient to justify testing. |
| Chaudhari et al. (95) | A pediatric perspective on genomics and prevention in the twenty-first century * | 2019 | Look at the unresolved questions which will benefit from future investigations of the role of genomics in disease prevention. | 7. N/A | N/A | N/A | N/A | N/A | The vast majority of these barriers cannot be adressed simply by making genomic sequencing faster or cheaper. Rather, they represent important choices societies need to make about the provision of public health services as well as clinical and public health informatics challenges. |
| Course & Hanks (96) | Newborn screening for cystic fibrosis: Is there benefit for everyone?* | 2019 | Address the role of NBS for CF - CFTR genetics and CF-SPID and false positives | 6. General public (NBS programs) + focus on patients (CF-SPID)/ families | N/A | N/A | N/A | N/A | NBS for CF has clearly improved the outcome for those with a ‘classical’ CF phenotype; however, CFTR becomes more complex and prognostication for some individuals becomes increasingly difficult, it is important to re-evaluate the role of newborn screening. |
| Dankert-Roelse & Vernooij-van Lamgen (97) | Newborn screening for cystic fibrosis: pros and cons* | 2011 | Review on argument in favor and against NBSCF | N/A | N/A | N/A | N/A | N/A | CFNBS leads to diagnosis earlier in life enabling correct healthcare management and treatment opportunities. |
| Farrell et al. (98) | Challenging the dogma of the healthy heterozygote: Implications for newborn screening policies and practices* | 2021 | Minireview providing an overview regarding health risk to carriers of AR conditions and discussing bioethical and policy implications for NBS | N/A | N/A | N/A | N/A | N/A | NBS programs of the public health system and medical care system have not been fully ready for the impact of the genetic information they generate. |
| Frankel et al. (99) | Potential Psychosocial Risks of Sequencing Newborns* | 2016 | review of psychological and genetic counseling literature of the factors at stake in gNBS | N/A | N/A | N/A | N/A | N/A | Possible psychosocial impacts of NBS exists |
| Grosse et al. (100) | Population Screening for Genetic Disorders in the 21st Century: Evidence, Economics, and Ethics* | 2010 | Review current evidence- based processes used (UK, USA, Netherlands) to assess genetic screening programs, including newborn screening programs, carrier screening, and organized cascade testing of relatives of patients with genetic syndromes. In particular, addressing critical evidentiary, economic, and ethical issues | N/A | N/A | N/A | N/A | N/A | Genetic screening policies have often been determined by technological capability, advocacy, and medical opinion rather than through a rigorous evidence-based review process. Decision making should take into account principles of ethics and opportunity costs. |
| Hasegawa et al. (101) | Parental Attitudes toward Ethical and Social Issues Surrounding the Expansion of Newborn Screening Using New Technologies* | 2011 | Assess parent knowledge of newborn screening (NBS) and parent attitudes toward NBS for untreatable conditions, NBS for late-onset disorders and informed consent | 4.Parents | 114 | all female | ≤20 4 (3.5%) 21–30 34 (29.8%) 31–40 59 (51.8%) 41–50 13 (11.4%) Blank 4 (3.5%) | USA | Parent attitudes differed from some in the medical community with regard to informed consent for NBS, NBS for conditions without effective treatments and predictive testing of children for late-onset disorders. |
| Hayeems et al. (102) | Expectations and values about expanded newborn screening: a public engagement study* | 2012 | Questioning the scope of NBS, and the role of parental choice | 1.General public | 60 | 60% female and 40% male | 27% of participants were 18–29 years old, 43% were 30–49, and 30% were >50 years old | Canada | Anticipated benefits of expanded infant screening were prioritized over harms, with information provision perceived as a mechanism for mitigating harms and enabling choice. |
| Howard et al. (103) | Whole-genome sequencing in newborn screening? A statement on the continued importance of targeted approaches in newborn screening programmes* | 2015 | To analyze the relevant issues specifically surrounding the potential use of genome sequencing in publicly funded NBS programmes and provide a set of recommendations | N/A | N/A | N/A | N/A | N/A | Discussion regarding the use of next-generation technology as a tool or the use of genome sequencing approaches in NBS raises questions about a possible paradigm shift in health care: will we use new sequencing technologies as a tool to answer focused clinical questions or will we use it to sequence entire genomes in order to return a set of results at birth and as a pure data and information generator, much of which can be analyzed and returned throughout a person’s lifetime? |
| Lantos (104) | Dangerous and expensive screening and treatment for rare childhood diseases: the case of Krabbe disease* | 2011 | Analysing the debate on NBS for rare diseases, using Krabbe as an example | N/A |  |  |  | USA | Expansion of NBS for rare disease beyond the Wilson and Junger criteria will need to see whether they can be implemented as a reasonable cost, and whether children actually benefit from early treatment. |
| Mak et al. (105) | Inborn errors of metabolism and expanded newborn screening: review and update* | 2013 | Literature review about recent expansion of NBS, its cost-effectiveness, associated pros and cons, and the ethical issues that can arise. Exposition of the analytical aspects of tandem mass spectrometry and post-analytical perspectives regarding result interpretation. | N/A | N/A | N/A | N/A | N/A | Despite the solid knowledge of improved technology, and savings achieved by the application of expended NBS in different countries, there are still numerous issues (ethical, technical and analytical) to be solved. |
| Miller et al. (106) | Clinical obligations and public health programmes: healthcare provider reasoning about managing the incidental results of newborn screening* | 2009 | Exploration of HCPs' beliefs/reasoning on the management of carrier status information generated through NBS, To inform policy on disclosure of infant sickle cell disorder (SCD) carrier results. | 2, HCPs | 1. survey - 1 615 2. interviews - 42 | N/A | N/A | Canada | A majority of respondents perceived a duty to disclose the incidental results of newborn screening, the policy implications of these attitudes are not obvious. Policy must balance descriptive ethics (i.e., what providers believe) and normative ethics (i.e., what duty-based principles oblige), address dissenting opinion and consider the relevance of moral principles grounded in clinical obligations for public health initiatives. |
| Reinstein (107) | Challenges of using next generation sequencing in newborn screening* | 2015 | Opinion This short article argues that there are medical, psychological, ethical and economic reasons why widespread dissemination of newborn screening is still premature. | N/A | N/A | N/A | N/A | Israel | Genetic screening in attractive but to premature to be implemented, A pre-testing consultation will have to be completed during pregnancy. Sessions should discuss the expectations of parents from their newborn’s genomic analyses, what information they would like to be disclosed, explanation of the meaning and significance of the screening results and at the same time ensuring that they understand it. When returning sequencing results, the genetic counsellors must give individualized attention to each clinical situation and to each family. They have to be familiar with the pre-test expectations of the parents and accordingly to counsel them based |
| Pollitt (108) | Different viewpoints: international perspectives on newborn screening* | 2015 | Review of different viewpoints on international perspectives on gNBS. | N/A | N/A | N/A | N/A | N/A | Current diversity of national policies relating to newborn screening reflects both practical limitations in health care organization and funding and widely different philosophical approaches to a variety of highly emotive issues. Decisions will become more difficult still as screening technology advances, with the use of orbitrap mass spectrometry for example, and the number and variety of disorders readily accessible increases still further. |
| Saich et al.(109) | Is Newborn Screening the Ultimate Strategy to Reduce Diagnostic Delays in Pompe Disease? The Parent and Patient Perspective* | 2020 | Providing an overview of the rationale for NBS from The Australian Pompe Association, its patients and their families | 6. Adults and Children | 1272 | 35% male, 65% female | 0–87 years | Australia | Delays in receiving a diagnosis are associated with anxiety, stress, symptomatic worsening, inappropriate use of resources and lack of access to appropriate support and care are still questioned. Health professional education is needed to increase awareness of rare diseases and improve the diagnostic process; Resources, are needed to support the requirements of people newly diagnosed with rare diseases. |
| Nicholls et al. (110) | Benefits and burdens of newborn screening: public understanding and decision-making* | 2014 | Review and summary of information on public and parental attitudes to the benefits and burdens of screening (expanded notions of benefits and burdens that have arisen as a result of increasing technological capabilities to identify underlying biological variation that has hitherto been impossible or impractical). | 6. General public and parents |  |  |  | N/A | Expanded notions of benefit and burden bring with them implications for parental consent and confidentiality and the secondary use of bloodspots. Important burden/benefit discussion identifying gaps in public and HCP understanding/attitudes/drivers towards NBS |
| Tassone (111) | Newborn Screening for Fragile X Syndrome* | 2014 | Analysis/review of benefits /drawbacks of the inclusion of FXS test in NBS screening given by an expert in the field | 6. genetic counselors, parents of a child with condition | 78946 | 90% male, 10% female | N/A | Taiwan, Canada, Spain, USA | Reasons to diagnose babies as carrying the premutation at the time of birth are similar to ones for those carrying mutations, including treatment and follow-up for the baby and benefits for family members. |
| Ulph & Bennett (112) | Psychological and Ethical Challenges of Introducing Whole Genome Sequencing into Routine Newborn Screening: Lessons Learned from Existing Newborn Screening* | 2022 | To ensure that parents are adequately informed in order that the consent they provide can be considered valid. | N/A | N/A | N/A | N/A | N/A | NBS programmes can reveal health information which may be useful in the future not the present (such as carrier status) or for which the current value is not known (uncertain diagnoses). This already raises serious challenges for parental consent, with parents deciding on behalf of their newborn whether this would be wanted – known as proxy consent. |

Note : * : the article did not used the exact term “genetic newborn screening” but the actual content of the articles fitted the scope of the analysis based on the inclusion and exclusion criteria

# VII. References

1. Lillie SE, Tarini BA, Janz NK, Zikmund-Fisher BJ. Framing optional genetic testing in the context of mandatory newborn screening tests. BMC medical informatics and decision making. 2015;15(1):1-7.

2. Temme R, Gruber A, Johnson M, Read L, Lu Y, McNamara J. Assessment of parental understanding of positive newborn screening results and carrier status for cystic fibrosis with the use of a short educational video. Journal of Genetic Counseling. 2015;24(3):473-81.

3. Christie L, Wotton T, Bennetts B, Wiley V, Wilcken B, Rogers C, et al. Maternal attitudes to newborn screening for fragile X syndrome. American Journal of Medical Genetics Part A. 2013;161(2):301-11.

4. Cyrus A, Street N, Quary S, Kable J, Kenneson A, Fernhoff P. Clinic-based infant screening for Duchenne muscular dystrophy: a feasibility study. PLoS Currents. 2012;4.

5. Davids L, Sun Y, Moore RH, Lisi E, Wittenauer A, Wilcox WR, et al. Health care practitioners' experience-based opinions on providing care after a positive newborn screen for Pompe disease. Mol Genet Metab. 2021;134(1-2):20-8.

6. Evans A, Lynch M, Johnson M, Bonhomme N. Assessing the newborn screening education needs of families living in medically underserved areas. Journal of Genetic Counseling. 2020;29(4):658-67.

7. Sands D. Frequency of high-quality communication behaviors used by primary care providers of heterozygous infants after newborn screening. Current Medical Literature. 2013;3(1):24.

8. Farrell MH, Sims AM, Kirschner ALP, Farrell PM, Tarini BA. Vulnerable child syndrome and newborn screening carrier results for cystic fibrosis or sickle cell. The journal of pediatrics. 2020;224:44-50. e1.

9. Farrell MH, Kirschner ALP, Tluczek A, Farrell PM. Experience with parent follow-up for communication outcomes after newborn screening identifies carrier status. The journal of pediatrics. 2020;224:37-43. e2.

10. Fitzgerald C, Linnane B, Heery E, Conneally N, George S, Fitzpatrick P. Newborn bloodspot screening for cystic fibrosis: what do antenatal and postnatal women know about cystic fibrosis? Journal of Cystic Fibrosis. 2016;15(4):436-42.

11. Genetti CA, Schwartz TS, Robinson JO, VanNoy GE, Petersen D, Pereira S, et al. Parental interest in genomic sequencing of newborns: enrollment experience from the BabySeq Project. Genetics in Medicine. 2019;21(3):622-30.

12. Hayeems RZ, Miller FA, Vermeulen M, Potter BK, Chakraborty P, Davies C, et al. False-positive newborn screening for cystic fibrosis and health care use. Pediatrics. 2017;140(5).

13. Karaceper MD, Chakraborty P, Coyle D, Wilson K, Kronick JB, Hawken S, et al. The health system impact of false positive newborn screening results for medium-chain acyl-CoA dehydrogenase deficiency: a cohort study. Orphanet journal of rare diseases. 2016;11:1-9.

14. Kasem A, Razeq NMA, Abuhammad S, Alkhazali H. Mothers’ knowledge and attitudes about newborn screening in Jordan. Journal of Community Genetics. 2022;13(2):215-25.

15. Nardini MD, Matthews AL, McCandless SE, Baumanis L, Goldenberg AJ. Genomic counseling in the newborn period: experiences and views of genetic counselors. Journal of genetic counseling. 2014;23:506-15.

16. Paquin RS, Peay HL, Gehtland LM, Lewis MA, Bailey Jr DB. Parental intentions to enroll children in a voluntary expanded newborn screening program. Social Science & Medicine. 2016;166:17-24.

17. Sadat R, Hall PL, Wittenauer AL, Vengoechea ED, Park K, Hagar AF, et al. Increased parental anxiety and a benign clinical course: Infants identified with short-chain acyl-CoA dehydrogenase deficiency and isobutyryl-CoA dehydrogenase deficiency through newborn screening in Georgia. Molecular Genetics and Metabolism. 2020;129(1):20-5.

18. Tluczek A, Orland KM, Cavanagh L. Psychosocial consequences of false-positive newborn screens for cystic fibrosis. Qualitative Health Research. 2011;21(2):174-86.

19. Vernooij-van Langen A, Van Der Pal S, Reijntjens A, Loeber J, Dompeling E, Dankert-Roelse J. Parental knowledge reduces long term anxiety induced by false-positive test results after newborn screening for cystic fibrosis. Molecular genetics and metabolism reports. 2014;1:334-44.

20. Wade CH, Elliott KR. Young adults’ attitudes toward pediatric whole-genome sequencing. Personalized Medicine. 2016;13(6):541-52.

21. Waisbren SE, Weipert CM, Walsh RC, Petty CR, Green RC. Psychosocial factors influencing parental interest in genomic sequencing of newborns. Pediatrics. 2016;137(Supplement_1):S30-S5.

22. Lang CW, Ross LF. Maternal attitudes about sickle cell trait identification in themselves and their infants. Journal of the National Medical Association. 2010;102(11):1065-72.

23. Weinreich SS, Rigter T, van El CG, Dondorp WJ, Kostense PJ, van der Ploeg AT, et al. Public support for neonatal screening for Pompe disease, a broad-phenotype condition. Orphanet journal of rare diseases. 2012;7(1):1-13.

24. Wood MF, Hughes SC, Hache LP, Naylor EW, Abdel‐Hamid HZ, Barmada MM, et al. Parental attitudes toward newborn screening for Duchenne/Becker muscular dystrophy and spinal muscular atrophy. Muscle & Nerve. 2014;49(6):822-8.

25. Wright SJ, Ulph F, Lavender T, Dharni N, Payne K. Understanding midwives’ preferences for providing information about newborn bloodspot screening. MDM Policy & Practice. 2018;3(1):2381468317746170.

26. Wright SJ, Ulph F, Dharni N, Payne K. Eliciting preferences for information provision in newborn bloodspot screening programs. Value in Health. 2017;20(4):651-61.

27. Farrell MH, Mooney KE, Laxova A, Farrell PM. Parental Preferences about Policy Options Regarding Disclosure of Incidental Genetic Findings in Newborn Screening: Using Videos and the Internet to Educate and Obtain Input. International Journal of Neonatal Screening. 2022;8(4):54.

28. Ong BAG, Osorio LMB, Ongtauco MA, Orosco GPC, Patrimonio DAD, Perez AD, et al. Knowledge, Attitudes, and Awareness Towards Newborn Screening in Association with Received Prenatal Care: A Survey of Primiparous Postpartum Mothers at the Philippine General Hospital. Acta Medica Philippina. 2022;56(7).

29. Peay HL, Gwaltney AY, Moultrie R, Cope H, Boyea BL, Porter KA, et al. Education and Consent for Population-Based DNA Screening: A Mixed-Methods Evaluation of the Early Check Newborn Screening Pilot Study. Front Genet. 2022;13:891592.

30. van der Pal SM, Wins S, Klapwijk JE, van Dijk T, Kater-Kuipers A, Van der Ploeg CP, et al. Parents’ views on accepting, declining, and expanding newborn bloodspot screening. Plos one. 2022;17(8):e0272585.

31. Wang X, Guan X-W, Wang Y-Y, Zhang Z-L, Li Y-H, Yang P-Y, et al. Current attitudes and preconceptions on newborn genetic screening in the Chinese reproductive-aged population. Orphanet Journal of Rare Diseases. 2022;17(1):1-10.

32. Wu X, Yang Y, Zhou L, Long W, Yu B. Are We Ready for Newborn Genetic Screening? A Cross-Sectional Survey of Healthcare Professionals in Southeast China. Frontiers in Pediatrics. 2022;10:875229.

33. Plass AM, van El CG, Pieters T, Cornel MC. Neonatal screening for treatable and untreatable disorders: prospective parents' opinions. Pediatrics. 2010;125(1):e99-106.

34. Pereira S, Smith HS, Frankel LA, Christensen KD, Islam R, Robinson JO, et al. Psychosocial effect of newborn genomic sequencing on families in the BabySeq Project: a randomized clinical trial. JAMA pediatrics. 2021;175(11):1132-41.

35. Oyeku SO, Feldman HA, Ryan K, Muret-Wagstaff S, Neufeld EJ. Primary care clinicians’ knowledge and confidence about newborn screening for sickle cell disease: randomized assessment of educational strategies. Journal of the National Medical Association. 2010;102(8):676-83.

36. Schwartz TS, Christensen KD, Uveges MK, Waisbren SE, McGuire AL, Pereira S, et al. Effects of participation in a US trial of newborn genomic sequencing on parents at risk for depression. Journal of genetic counseling. 2022;31(1):218-29.

37. Carlton J, Griffiths H, Horwood A, Mazzone P, Walker R, Simonsz H. Acceptability of childhood screening: a systematic narrative review. Public Health. 2021;193:126-38.

38. Tluczek A, Ersig AL, Lee S. Psychosocial issues related to newborn screening: a systematic review and synthesis. International Journal of Neonatal Screening. 2022;8(4):53.

39. Clark CC, Boardman FK. Expanding the notion of “benefit”: Comparing public, parent, and professional attitudes towards whole genome sequencing in newborns. New Genetics and Society. 2022;41(2):96-115.

40. Acharya K, Lang CW, Ross LF. A pilot study to explore knowledge, attitudes, and beliefs about sickle cell trait and disease. Journal of the National Medical Association. 2009;101(11):1163-72.

41. Acharya K, Ross LF. Fragile X screening: attitudes of genetic health professionals. American Journal of Medical Genetics Part A. 2009;149(4):626-32.

42. Acharya K, Schindler A. Developmental and behavioral pediatricians' attitudes toward screening for fragile X. American journal on intellectual and developmental disabilities. 2013;118(4):284-93.

43. Araia MH, Wilson BJ, Chakraborty P, Gall K, Honeywell C, Milburn J, et al. Factors associated with knowledge of and satisfaction with newborn screening education: a survey of mothers. Genetics in medicine. 2012;14(12):963-70.

44. Pruniski B, Lisi E, Ali N. Newborn screening for Pompe disease: Impact on families. Journal of inherited metabolic disease. 2018;41:1189-203.

45. Bailey Jr DB, Bishop E, Raspa M, Skinner D. Caregiver opinions about fragile X population screening. Genetics in Medicine. 2012;14(1):115-21.

46. Boardman FK, Sadler C, Young PJ. Newborn genetic screening for spinal muscular atrophy in the UK: The views of the general population. Molecular genetics & genomic medicine. 2018;6(1):99-108.

47. Boardman FK. Attitudes toward population screening among people living with fragile X syndrome in the UK:‘I wouldn’t wish him away, I’d just wish his fragile X syndrome away’. Journal of Genetic Counseling. 2021;30(1):85-97.

48. Bombard Y, Miller FA, Hayeems RZ, Barg C, Cressman C, Carroll JC, et al. Public views on participating in newborn screening using genome sequencing. European Journal of Human Genetics. 2014;22(11):1248-54.

49. Bukini D, Nkya S, McCurdy S, Mbekenga C, Manji K, Parker M, et al. Perspectives on building sustainable newborn screening programs for sickle cell disease: experience from Tanzania. International Journal of Neonatal Screening. 2021;7(1):12.

50. Burlina AB, Corsello G. Survey of Italian pediatricians’ perspectives and knowledge about neonatal screening. Italian journal of pediatrics. 2015;41:1-4.

51. Cao M, Notini L, Ayres S, Vears DF. Australian healthcare professionals' perspectives on the ethical and practical issues associated with genomic newborn screening. Journal of Genetic Counseling. 2023;32(2):376-86.

52. Chudleigh J, Chinnery H, Bonham JR, Olander E, Moody L, Simpson A, et al. Qualitative exploration of health professionals’ experiences of communicating positive newborn bloodspot screening results for nine conditions in England. BMJ open. 2020;10(10):e037081.

53. Chudleigh J, Buckingham S, Dignan J, O’Driscoll S, Johnson K, Rees D, et al. Parents’ experiences of receiving the initial positive newborn screening (NBS) result for cystic fibrosis and sickle cell disease. Journal of genetic counseling. 2016;25:1215-26.

54. Conway M, Vuong TT, Hart K, Rohrwasser A, Eilbeck K. Pain points in parents’ interactions with newborn screening systems: a qualitative study. BMC pediatrics. 2022;22(1):167.

55. Crossen K, Berry L, Myers MF, Leslie N, Goueli C. A qualitative study: mothers’ experiences of their child’s late-onset Pompe disease diagnosis following newborn screening. International Journal of Neonatal Screening. 2022;8(3):43.

56. DeLuca JM, Kearney MH, Norton SA, Arnold GL. Parents' experiences of expanded newborn screening evaluations. Pediatrics. 2011;128(1):53-61.

57. Jessup M, Douglas T, Priddis L, Branch-Smith C, Shields L. Parental experience of information and education processes following diagnosis of their infant with cystic fibrosis via newborn screening. Journal of pediatric nursing. 2016;31(3):e233-e41.

58. Johnson F, Southern KW, Ulph F. Psychological impact on parents of an inconclusive diagnosis following newborn bloodspot screening for cystic fibrosis: a qualitative study. International journal of neonatal screening. 2019;5(2):23.

59. Joseph G, Chen F, Harris-Wai J, Puck JM, Young C, Koenig BA. Parental views on expanded newborn screening using whole-genome sequencing. Pediatrics. 2016;137(Supplement_1):S36-S46.

60. Kusyk D, Acharya K, Garvey K, Ross LF. A pilot study to evaluate awareness of and attitudes about prenatal and neonatal genetic testing in postpartum African American women. Journal of the National Medical Association. 2013;105(1):85-91.

61. La Pean A, Collins JL, Christopher SA, Eskra KL, Roedl SJ, Tluczek A, et al. A qualitative secondary evaluation of statewide follow-up interviews for abnormal newborn screening results for cystic fibrosis and sickle cell hemoglobinopathy. Genetics in medicine. 2012;14(2):207-14.

62. Lang CW, Stark AP, Acharya K, Ross LF. Maternal knowledge and attitudes about newborn screening for sickle cell disease and cystic fibrosis. American Journal of Medical Genetics Part A. 2009;149(11):2424-9.

63. Leppert K, Bisordi K, Nieto J, Maloney K, Guan Y, Dixon S, et al. Genetic counselors’ experience with and opinions on the management of newborn screening incidental carrier findings. Journal of genetic counseling. 2018;27(6):1328-40.

64. Lipstein EA, Nabi E, Perrin JM, Luff D, Browning MF, Kuhlthau KA. Parents' decision-making in newborn screening: opinions, choices, and information needs. Pediatrics. 2010;126(4):696-704.

65. Lisi EC, McCandless SE. Newborn screening for lysosomal storage disorders: views of genetic healthcare providers. Journal of genetic counseling. 2016;25:373-84.

66. Mak CM, Law EC, Lee HH, Siu W, Chow K, Au Yeung S, et al. The first pilot study of expanded newborn screening for inborn errors of metabolism and survey of related knowledge and opinions of health care professionals in Hong Kong. Hong Kong Medical Journal. 2018.

67. Noke M, Ulph F. Young adults’ pre-existing knowledge of cystic fibrosis and sickle cell diseases: implications for newborn screening. Journal of genetic counseling. 2014;23:121-30.

68. Miller FA, Paynter M, Hayeems RZ, Little J, Carroll JC, Wilson BJ, et al. Understanding sickle cell carrier status identified through newborn screening: a qualitative study. European journal of human genetics. 2010;18(3):303-8.

69. Moody L, Atkinson L, Kehal I, Bonham JR. Healthcare professionals’ and parents’ experiences of the confirmatory testing period: a qualitative study of the UK expanded newborn screening pilot. BMC pediatrics. 2017;17:1-10.

70. Nnodu OE, Adegoke SA, Ezenwosu OU, Emodi II, Ugwu NI, Ohiaeri CN, et al. A multi-centre survey of acceptability of newborn screening for sickle cell disease in Nigeria. Cureus. 2018;10(3).

71. Peterson L, Siemon A, Olewiler L, McBride KL, Allain DC. A qualitative assessment of parental experiences with false‐positive newborn screening for Krabbe disease. Journal of Genetic Counseling. 2022;31(1):252-60.

72. Prakash S, Penn JD, Jackson KE, Dean LW. Newborn screening for Pompe disease: Parental experiences and follow‐up care for a late‐onset diagnosis. Journal of Genetic Counseling. 2022;31(6):1404-20.

73. Raspa M, Lynch M, Squiers L, Gwaltney A, Porter K, Peay H, et al. Information and emotional support needs of families whose infant was diagnosed with SCID through newborn screening. Frontiers in Immunology. 2020;11:524796.

74. Salm N, Yetter E, Tluczek A. Informing parents about positive newborn screen results: parents' recommendations. Journal of Child Health Care. 2012;16(4):367-81.

75. Perobelli S, Zanolla L, Tamanini A, Rizzotti P, Maurice Assael B, Castellani C. Inconclusive Cystic Fibrosis neonatal screening results: long‐term psychosocial effects on parents. Acta paediatrica. 2009;98(12):1927-34.

76. Sims AM, Cromartie SJ, Gessner L, Campbell A, Coker T, Wang CJ, et al. Parents’ experiences and needs regarding infant sickle cell trait results. Pediatrics. 2022;149(5):e2021053454.

77. Stark AP, Lang CW, Ross LF. A pilot study to evaluate knowledge and attitudes of Illinois pediatricians toward newborn screening for sickle cell disease and cystic fibrosis. American journal of perinatology. 2010:169-76.

78. Nicholls SG, Southern KW. Parental decision-making and acceptance of newborn bloodspot screening: an exploratory study. PLoS One. 2013;8(11):e79441.

79. Tarini BA, Clark SJ, Pilli S, Dombkowski KJ, Korzeniewski SJ, Gebremariam A, et al. False-positive newborn screening result and future health care use in a state Medicaid cohort. Pediatrics. 2011;128(4):715-22.

80. Timmins GT, Wynn J, Saami AM, Espinal A, Chung WK. Diverse parental perspectives of the social and educational needs for expanding newborn screening through genomic sequencing. Public Health Genomics. 2022;25(5-6):185-92.

81. Tluczek A, McKechnie AC, Brown RL. Factors associated with parental perception of child vulnerability 12 months after abnormal newborn screening results. Research in nursing & health. 2011;34(5):389-400.

82. Tu W-J, He J, Chen H, Shi X-D, Li Y. Psychological effects of false-positive results in expanded newborn screening in China. PLoS One. 2012;7(4):e36235.

83. Ulph F, Cullinan T, Qureshi N, Kai J. Familial influences on antenatal and newborn haemoglobinopathy screening. Ethnicity & health. 2011;16(4-5):361-75.

84. van Dijk T, Kater A, Jansen M, Dondorp WJ, Blom M, Kemp S, et al. Expanding neonatal bloodspot screening: a multi-stakeholder perspective. Frontiers in pediatrics. 2021;9:706394.

85. Vansenne F, de Borgie CA, Legdeur M, Spauwen MO, Peters M. Providing genetic risk information to parents of newborns with sickle cell trait: role of the general practitioner in neonatal screening. Genetic Testing and Molecular Biomarkers. 2011;15(10):671-5.

86. Koopmans J, Ross LF. Identification and management of sickle cell trait by young physicians. Journal of the National Medical Association. 2012;104(5-6):299-304.

87. Hayeems RZ, Miller FA, Carroll JC, Little J, Allanson J, Bytautas JP, et al. Primary care role in expanded newborn screening: After the heel prick test. Canadian Family Physician. 2013;59(8):861-8.

88. Hayeems RZ, Miller FA, Barg CJ, Bombard Y, Chakraborty P, Potter BK, et al. Primary care providers’ role in newborn screening result notification for cystic fibrosis. Canadian Family Physician. 2021;67(6):439-48.

89. Goldenberg AJ, Ponsaran R, Gaviglio A, Simancek D, Tarini BA. Genomics and newborn screening: Perspectives of public health programs. International Journal of Neonatal Screening. 2022;8(1):11.

90. Bukini D, Mbekenga C, Nkya S, Malasa L, McCurdy S, Manji K, et al. Influence of gender norms in relation to child’s quality of care: follow-up of families of children with SCD identified through NBS in Tanzania. Journal of Community Genetics. 2021;12:143-54.

91. Azzopardi PJ, Upshur R, Luca S, Venkataramanan V, Potter BK, Chakraborty PK, et al. Health-care providers’ perspectives on uncertainty generated by variant forms of newborn screening targets. Genetics in Medicine. 2020;22(3):566-73.

92. Bailey Jr DB, Wheeler A, Berry-Kravis E, Hagerman R, Tassone F, Powell CM, et al. Maternal consequences of the detection of fragile X carriers in newborn screening. Pediatrics. 2015;136(2):e433-e40.

93. Blom M, Schoenaker MH, Hulst M, De Vries MC, Weemaes CM, Willemsen MA, et al. Dilemma of reporting incidental findings in newborn screening programs for SCID: parents’ perspective on ataxia telangiectasia. Frontiers in Immunology. 2019;10:2438.

94. Botkin JR. Ethical issues in pediatric genetic testing and screening for current opinion in pediatrics. Current Opinion in Pediatrics. 2016;28(6):700.

95. Chaudhari BP, Manickam K, McBride KL. A pediatric perspective on genomics and prevention in the twenty-first century. Pediatric Research. 2020;87(2):338-44.

96. Course C, Hanks R. Newborn screening for cystic fibrosis: Is there benefit for everyone? Paediatric Respiratory Reviews. 2019;31:3-5.

97. Dankert-Roelse J, Vernooij-van Langen A. Newborn screening for cystic fibrosis: pros and cons. Breathe. 2011;8(1):24-30.

98. Farrell PM, Langfelder-Schwind E, Farrell MH. Challenging the dogma of the healthy heterozygote: Implications for newborn screening policies and practices. Molecular Genetics and Metabolism. 2021;134(1-2):8-19.

99. Frankel LA, Pereira S, McGuire AL. Potential psychosocial risks of sequencing newborns. Pediatrics. 2016;137(Supplement_1):S24-S9.

100. Grosse S, Rogowski W, Ross L, Cornel M, Dondorp W, Khoury M. Population screening for genetic disorders in the 21st century: evidence, economics, and ethics. Public health genomics. 2009;13(2):106-15.

101. Hasegawa L, Fergus K, Ojeda N, Au S. Parental attitudes toward ethical and social issues surrounding the expansion of newborn screening using new technologies. Public health genomics. 2011;14(4-5):298-306.

102. Hayeems RZ, Miller FA, Bombard Y, Avard D, Carroll J, Wilson B, et al. Expectations and values about expanded newborn screening: a public engagement study. Health expectations. 2015;18(3):419-29.

103. Howard HC, Knoppers BM, Cornel MC, Wright Clayton E, Sénécal K, Borry P. Whole-genome sequencing in newborn screening? A statement on the continued importance of targeted approaches in newborn screening programmes. European journal of human genetics. 2015;23(12):1593-600.

104. Lantos JD. Dangerous and expensive screening and treatment for rare childhood diseases: the case of Krabbe disease. Developmental disabilities research reviews. 2011;17(1):15-8.

105. Mak CM, Lee H-CH, Chan AY-W, Lam C-W. Inborn errors of metabolism and expanded newborn screening: review and update. Critical reviews in clinical laboratory sciences. 2013;50(6):142-62.

106. Miller F, Hayeems R, Bombard Y, Little J, Carroll J, Wilson B, et al. Clinical obligations and public health programmes: healthcare provider reasoning about managing the incidental results of newborn screening. Journal of medical ethics. 2009;35(10):626-34.

107. Reinstein E. Challenges of using next generation sequencing in newborn screening. Genetics Research. 2015;97:e21.

108. Pollitt RJ. Different viewpoints: International perspectives on newborn screening. Journal of Medical Biochemistry. 2015;34(1):18.

109. Saich R, Brown R, Collicoat M, Jenner C, Primmer J, Clancy B, et al. Is newborn screening the ultimate strategy to reduce diagnostic delays in Pompe disease? The parent and patient perspective. International journal of neonatal screening. 2020;6(1):1.

110. Nicholls SG, Wilson BJ, Etchegary H, Brehaut JC, Potter BK, Hayeems R, et al. Benefits and burdens of newborn screening: public understanding and decision-making. Personalized Medicine. 2014;11(6):593-607.

111. Tassone F. Newborn screening for fragile X syndrome. JAMA neurology. 2014;71(3):355-9.

112. Ulph F, Bennett R. Psychological and ethical challenges of introducing whole genome sequencing into routine newborn screening: lessons learned from existing newborn screening. The New Bioethics. 2023;29(1):52-74.
