# Supplementary material for: Preferences, attitudes and views regarding genetic newborn screening (gNBS) for rare diseases: a systematic review of the literature and synthesis from 2009 to 2022
Source: Orphanet J Rare Dis. 2026 Jan 8;21:27. doi: 10.1186/s13023-025-04179-0 (PMC12836846; doi:10.1186/s13023-025-04179-0)
Supplement: Supplementary file 5 — Supplementary Material 5 [file 13023_2025_4179_MOESM5_ESM.docx]

| **Appendix 2** : Articles themes | **Title** | **Year** | **Opportunities:  1-Early diagnosis and early treatment or interventionà reduce the cost and burden of diagnostic odyssey 2-Family planning 3-Understanding and documentation of the health status of the child for the future 4-Improved healthcare: (advance in knowledge and science) 5-Improved health behaviors/better quality of life/ better chance at an improved life (improvement on the patient/family level) 6-Support early diagnosis for untreatable conditions (non-medical benefits) 7- Understanding and acceptance of the disease state 8-Not discussed or not captured/ Not applicable (N/A)  (JA, SM, GK)** | **Barriers:  1-Impact on relation with the child and intra-familial relationships 2-Consequences of carrier status 3-Misunderstanding of shared information or lack of it (Education and communication strategies) 4-Not meeting predefined criteria for NBS programs/actionability 5-Burden of being part of a study/ burden of cost and logistics 6-Fears of discrimination and privacy concerns  7-Risk of false results or diagnostic uncertainty or unwanted information à stress 9-Religious beliefs  10-Various and psycho-social implications** | **Attitudes:  1-Positive attitudes: opportunity for early diagnosis, family planning, prepare for a baby’s needs, future improvement in quality of life. Education is key 2-Positive attitude linked to actionability 3-Attitude varied according to shared information/education  4-Not discussed or not captured 5-Negative attitudes: concerns for logistics, anxiety regarding results, no interest in research, avoiding harm, religious beliefs, insufficient knowledge 6-Mixed  7-Support among parents and general public 8-Support among HCPs** |
| --- | --- | --- | --- | --- | --- |
| Acharya | A Pilot Study to explore Knowledge, Attitudes and Beliefs about Sickle Cell Trait and Disease | 2009 | 2, 6 | 2,3,10 | 4 |
| Acharya K and Ross L | Fragile X Screening: Views of Genetic Health Professionals | 2009 | 2,5 | 4,10 | 8 |
| Acharya K and Schindler A | Developmental and Behavioral Pediatricians Attitudes Toward Screening for Fragile X | 2013 | 5 | 2, 3, 4,1,10+ impact on future prenancies | 1,2 |
| Plass | Neonatal Screening for Treatable and Untreatable Disorders: Prospective Parents’ Opinions | 2009 | 1, 2 | 4, 5 | 1, 2 |
| Araia | Factors associated with knowledge of and satisfaction with newborn screening education: a survey of mothers | 2012 | 1 | 3 | 4 |
| Azzopardi P | Health-care providers’ perspectives on uncertainty generated by variant forms of newborn screening targets | 2020 | 6 | 7 | 4 |
| Pruniski | Newborn screening for Pompe disease: impact on families | 2018 | 2,4 | 1, 2, 3, 5, 6, 7, 10 | 1, 2 |
| Bailey | Caregiver Opinions about Fragile X Population Screening | 2012 | 2, 3,1 | 10,1, 2, 3, 4,6 | 1,2 |
| Bailey | Maternal Consequences of the Detection of Fragile X Carriers in Newborn Screening | 2015 | 1, 2 | 2, 4 | 4 |
| Blom | Dilemma of Reporting incidental findings in newborn screening programs for SCID: parents perspective on ataxia telangiectasia | 2019 | 5,1,2,3 | 1,7 | 1 |
| Boardman | newborn genetic screening for spinal muscular atrophy in the UK: the views of the general population | 2017 | 1,2, 5 | 1,10 | 1,2,7 |
| Boardman | Attitudes toward population screening among people living with fragile X syndrome in the UK:'I wouldn't wish him away, I'd just wish his frigile X syndrome away' | 2020 | 1,2, | 5, 6 | 6,2 |
| Bombard | Public views on participating in newborn screening using genome sequencing | 2014 | 4 | 3 | 1, 7 |
| Botkin | Ethical issues in pediatric genetic testing and screening for current opinion in pediatrics | 2017 | **2, 7** | 4, 6, 7, 10 |  |
| Bukini | Perspectives on Building Sustainable Newborn Screening Programs for Sickle Cell Disease: Experience from Tanzania | 2021 | 4 | 5,3 | 1 |
| Bukini | Influence of gender norms in relation to child’s quality of care: follow-up of families of children with SCD identified through NBS in Tanzania | 2021 | 4 | 1,3,10 | 6,4 |
| Burlina | Survey of Italian pediatricians’ perspectives and knowledge about neonatal screening | 2015 | 1 | 5,7 | 1, 2, |
| Cao | Australian healthcare professionals' perspectives on the ethical and practical issues associated with genomic newborn screening | 2022 | 1,2 | 7,6 | 1 |
| Carlton | Acceptability of childhood screening: a systematic narrative review | 2021 | 6 | 5, 7, 10 | 6 |
| Chaudhari | A pediatric perspective on genomics and prevention in the twenty-first century | 2019 | **4, 5** | 3, 5, 6, 7, 10 |  |
| Christie | Maternal Attitudes to Newborn Screening for Fragile X Syndrome | 2013 | 1, 2, 3, 4, 5 | 1, 2, 7 | 1 |
| Chudleigh | Qualitative exploration of health professionals’ experiences of communicating positive newborn bloodspot screening results for nine conditions in England | 2020 | 6 | 5, 10 | 4 |
| Chudleigh | Parents’ Experiences of Receiving the Initial Positive Newborn Screening (NBS) Result for Cystic Fibrosis and Sickle Cell Disease | 2016 | 4,1, 2, 5 | 1, 3, 7, 10 | 4 |
| Clark | Expanding the notion of “benefit”: comparing public, parent, and professional attitudes towards whole genome sequencing in newborns | 2022 | 1, 2, 3, 4 | 1, 2, 3, 5, 6, 7, 10 | 6, **7, 8,** |
| Conway | Pain points in parents’ interactions with newborn screening systems: a qualitative study | 2022 | 1 | 5, 10 | 6,1 |
| Course | Newborn screening for cystic fibrosis: Is there benefit for everyone? | 2019 | 1 | 2, 5, 6, 7, 10 |  |
| Crossen | A Qualitative Study: Mothers’ Experiences of Their Child’s Late-Onset Pompe Disease Diagnosis Following Newborn Screening | 2022 | 5 | 3,7, 10 | 5 |
| Cyrus | Clinic based infant screening for Duchenne Muscular Dystrophy: A feasibility study | 2012 | 1, 2, 5 | 1, 3, 4, 7 | 1, 2, 7, 8 |
| Dankert-Roelse | Newborn screening for cystic fibrosis: pros and cons | 2011 | 1, 2, 4, 5, 6 | 2, 6, 7, 10 |  |
| Davids | Health care practitioners' experience-based opinions on providing care after a positive newborn screen for Pompe disease | 2021 | 1, 2, 5 | 1, 3, 4, 5, 6. 7, 10 | 3, 6, 8 |
| De Luca | Parents' experiences of expanded newborn screening | 2011 | 6 | 10, 3, 7 | 1 |
| Evans A | Assessing the newborn screening education needs of families living in medically underserved areas | 2020 | 6 | 3, 10 | 4 |
| Farrell M | Frequency of high-quality communication behaviors used by primary care providers of heterozygous infants after newborn screening | 2013 | 6 | 1, 2, **3**, 10 | 4 |
| Farrell M | Parental Preferences about Policy Options Regarding Disclosure of Incidental Genetic Findings in Newborn Screening: Using Videos and the Internet to Educate and Obtain Input | 2022 | 6 | 7, 10 | 4 |
| Farrell MH | Vulnerable Child Syndrome and Newborn Screening Carrier Results for Cystic Fibrosis or Sickle Cell | 2020 | 6 | 1, 2, 7 | 4 |
| Farrell MH | Experience with Parent Follow-Up for Communication Outcomes after Newborn Screening Identifies Carrier Status | 2020 | 6 | 2, 3, 7, 10 | 5 |
| Farrell | Challenging the dogma of the healthy heterozygote: Implications for newborn screening policies and practices | 2021 | 1, 5 | 2, 3, 7, 10 |  |
| Fitzgerald | Newborn bloodspot screening for cystic fibrosis: What do antenatal and postnatal women know about cystic fibrosis? | 2016 | 6 | 2, 3 | 4 |
| Frankel | Potential Psychosocial Risks of Sequencing Newborns | 2016 | 8 | 1, 2, 7, 10 |  |
| Genetti | Parental interest in genomic sequencing of newborns: enrollent experience from the BabySeq project | 2019 | 1 | 5, 6, 7, 10 | 5 |
| Goldenberg | Genomics and Newborn Screening: Perspectives of Public Health Programs | 2022 | 4, 5 | 5, 3,7 | 6 |
| Grosse | Population Screening for Genetic Disorders in the 21st Century: Evidence, Economics, and Ethics | 2010 | 1, 2 | 2, 3, 6, 7, 10 |  |
| Hasegawa | Parental Attitudes toward Ethical and Social Issues Surrounding the Expansion of Newborn Screening Using New Technologies | 2011 | 1, 2, 3, 4, 6, 7 | 1, 3, 4, 10 |  |
| Hayeems | Expectations and values about expanded newborn screening: a public engagement study | 2012 | 1, 2, 4, 5, 6 | 1, 2, 3, 4, 6, 7, 10 |  |
| Hayeems | False-Positive Newborn Screening for Cystic Fibrosis and Health Care Use | 2017 | 6 | 10 | 4 |
| Hayeems | Primary care role in expanded newborn screening | 2013 | 6 | 3, 5 | 4 |
| Hayeems | Primary care providers’ role in newborn screening result notification for cystic fibrosis | 2021 | 6 | 3.5 | 4 |
| Howard | Whole-genome sequencing in newborn screening? A statement on the continued importance of targeted approaches in newborn screening programmes | 2015 | 4 | 1, 3, 4, 5, 7, 10 |  |
| Jessup | Parental Experience of Information and Education Processes Following Diagnosis of Their Infant With Cystic Fibrosis Via Newborn Screening | 2016 | 1,5 | 7,5, 10 | 4 |
| Johnson | Psychological Impact on Parents of an Inconclusive Diagnosis Following Newborn Bloodspot Screening for Cystic Fibrosis: A Qualitative Study | 2019 | 3 | 3, 5, 7, 10 | 6 |
| Joseph | Parental Views on Expanded Newborn Screening Using Whole-Genome Sequencing | 2016 | 4,3, 1 | 5, 6, 7, 10 | 6 |
| Karaceper M | The health system impact of false positive newborn screening results for medium-chain acyl-CoA dehydrogenase deficiency: a cohort study | 2016 | 1, 5 | 5, 7 | 4 |
| Kasem A | Mothers’ knowledge and attitudes about newborn screening in Jordan | 2022 | 1, 5 | 3, 6, 10 | 1, 2 |
| Koopmans | Identification and Management of Sickle Cell Trait by Young Physicians | 2012 | 6 | 3 | 4 |
| Kusyk | A Pilot Study to Evaluate Awareness of and Attitudes About Prenatal and Neonatal Genetic Testing in Postpartum African American Women | 2013 | 2 | 3 | 4 |
| La Pean | A qualitative secondary evaluation of statewide follow-up interviews for abnormal newborn screening results for cystic fibrosis and sickle cell hemoglobinopathy | 2012 | 3, 1, 5 | 2, 3, 10 | 1,2 |
| Lang | Maternal Knowledge and Attitudes About Newborn Screening for Sickle Cell Disease and Cystic Fibrosis | 2009 | 4 | 3 | 1,2 |
| Lantos | DANGEROUS AND EXPENSIVE SCREENING AND TREATMENT FOR RARE CHILDHOOD DISEASES: THE CASE OF KRABBE DISEASE | 2011 | 2, 3, 4, 6 | 5, 7, 10 |  |
| Leppert | Genetic Counselors’ Experience with and Opinions on the Management of Newborn Screening Incidental Carrier Findings | 2018 | 3,4,2 | 2, 5, 7, 10 | 1,3 |
| Lillie S | Framing optional genetic testing in the context of mandatory newborn screening tests | 2015 | 6 | 3 | 3, 6 |
| Lipstein | Parents’ Decision-Making in Newborn Screening: Opinions, Choices, and Information Needs | 2010 | 1, 2, 3, 4 | 5, 6, 7, 10 | 1,2,3 |
| Lisi | Newborn Screening for Lysosomal Storage Disorders: Views of Genetic Healthcare Providers | 2016 | 1, 2 | 4, 10 | 1,6 |
| Mak | Inborn errors of metabolism and expanded newborn screening: review and update | 2013 | 1, 2, ,4 | 2, 3, 4, 5, 6, 7, 10 |  |
| Mak | The first pilot study of expanded newborn screening for inborn errors of metabolism and survey of related knowledge and opinions of health care professionals in Hong Kong | 2018 | 1 | 3 | 8 |
| Noke | Young Adults’ Pre-Existing Knowledge of Cystic Fibrosis and Sickle Cell Diseases: Implications for Newborn Screening | 2013 | 6 | 2,3 | 4 |
| Miller | Clinical obligations and public health programmes: healthcare provider reasoning about managing the incidental results of newborn screening | 2009 | 2 | 2, 3, 7 |  |
| Miller | Understanding sickle cell carrier status identified through newborn screening: a qualitative study | 2010 | 1, 2, 5 | 2,3,7 | 4 |
| Moody | Healthcare professionals’ and parents’ experiences of the confirmatory testing period: a qualitative study of the UK expanded newborn screening pilot | 2017 | 1, 4 | 3,5, 7 | 1,6 |
| Nardini M | Genomic Counseling in the Newborn Period: Experiences and Views of Genetic Counselors | 2014 | 1, 4, 5 | 3, 5, 7, 10 | 5 |
| Nnodu | A Multi-centre Survey of Acceptability of Newborn Screening for Sickle Cell Disease in Nigeria | 2018 | 4 | 3, 5, 9 | 1 |
| Ong | Knowledge, Attitudes, and Awareness Towards Newborn Screening in Association with Received Prenatal Care: A Survey of Primiparous Postpartum Mothers at the Philippine General Hospital | 2022 | 1 | 1, 3, 7, 10 | 1, 2 |
| Oyeku | Primary Care Clinicians’ Knowledge and Confidence About Newborn Screening for Sickle Cell Disease: Randomized Assessment of Educational Strategies | 2010 | 6 | 3, 5 | 4 |
| Peay | Education and Consent for Population-Based DNA Screening: A Mixed-Methods Evaluation of the Early Check Newborn Screening Pilot Study | 2022 | 1, 3, 4 | 3, 6 | 1, 2 |
| Pereira | Psychosocial Effect of Newborn Genomic Sequencing on Families in the BabySeq Project A Randomized Clinical Trial | 2021 | 1, 2, 4, 5 | 1, 10 | 4 |
| Peterson | A qualitative assessment of parental experiences with false-positive newborn screening for Krabbe disease | 2022 | 1,3 | 7,10,3 | 1 |
| Prakash | Newborn screening for Pompe disease: Parental experiences and follow-up care for a late-onset diagnosis | 2022 | 1 | 3,10,7, | 3 |
| Raspa M. | Information and Emotional Support Needs of Families Whose Infant Was Diagnosed With SCID Through Newborn Screening | 2020 | 1 | 3,10 need for support | 4 |
| Reinstein | Challenges of using next generation sequencing in newborn screening | 2015 | 8 | 1, 2, 5, 6, 7, 10 |  |
| Pollitt | DIFFERENT VIEWPOINTS: INTERNATIONAL PERSPECTIVES ON NEWBORN SCREENING | 2015 | 1 | 2, 3, 4, 5, 6, 10 |  |
| Paquin | Parental intentions to enroll children in a voluntary expanded newborn screening program | 2016 | 1, 2, 3, 5 | 3 | 1, 2 |
| Sadat | Increased parental anxiety and a benign clinical course: Infants identified with short-chain acyl-CoA dehydrogenase deficiency and isobutyryl-CoA dehydrogenase deficiency through newborn screening in Georgia | 2020 | 4 | 7, 10 | 4 |
| Saich | Is Newborn Screening the Ultimate Strategy to Reduce Diagnostic Delays in Pompe Disease? The Parent and Patient Perspective | 2020 | 1, 2, 5 | 3, 5, 10 |  |
| Salm | Informing parents about positive newborn screen results: Parents’ recommendations | 2012 | 6 | 10, 1 | 4 |
| Perobelli | Inconclusive Cystic Fibrosis neonatal screening results: long-term psychosocial effects on parents | 2009 | 1, 2, 5 | 10,7 | 2 |
| Schwartz | Effects of participation in a U.S. trial of newborn genomic sequencing on parents at risk for depression | 2021 | 4 | 7, 10 | 4 |
| Sims | Parents’ Experiences and Needs Regarding Infant Sickle Cell Trait Results | 2022 | 2,3 | 3,2, 10 | 4 |
| Stark | A Pilot Study to Evaluate Knowledge and Attitudes of Illinois Pediatricians toward Newborn Screening for Sickle Cell Disease and Cystic Fibrosis | 2011 | 6 | 3, 5, 7 | 1, 8 |
| Nicholls | Benefits and burdens of newborn screening: public understanding and decision-making | 2014 | 1, 2, 3, 5 | 3, 5, 6, 7, 10 |  |
| Nicholls | Parental Decision-Making and Acceptance of Newborn Bloodspot Screening: An Exploratory Study | 2013 | 1 | 3, 5, 10 | 3 Seven factors were identified as being either explicitly or implicitly related to parental decision-making: Experience, Attitudes to medicine, Information-seeking behaviour, Perceived knowledge, Attitudes to screening, and Perceived choice, all of which ultimately impact on Perceived decisional quality. |
| Tarini | False-Positive Newborn Screening Result and Future Health Care Use in a State Medicaid Cohort | 2011 | 6 | 7, 10 | 4 |
| Tassone | Newborn Screening for Fragile X Syndrome | 2014 | 1, 2, 5, 6 | 2, 3, 4, 5, 7 |  |
| Temme R. | Assessment of Parental Understanding of Positive Newborn Screening Results and Carrier Status for Cystic Fibrosis with the use of a Short Educational Video | 2015 | 6 | 3 | 4 |
| Timmins | Diverse Parental Perspectives of the Social and Educational Needs for Expanding Newborn Screening through Genomic Sequencing | 2022 | 3, 4, 5 | 3, 6, 7, 10 | 1,2 |
| Tluczek | Psychosocial Issues Related to Newborn Screening: A Systematic Review and Synthesis | 2022 | 1, 2, 5 | 1, 2, 3, 4, 6, 7, 10 | 6 |
| Tluczek | Psychosocial Consequences of False-Positive Newborn Screens for Cystic Fibrosis | 2011 | 1, 2, 3, 5 | 1, 2, 3, 5, 7, 10 | 1, 2 |
| Tluczek | Factors Associated With Parental Perception of Child Vulnerability 12 Months After Abnormal Newborn Screening Results | 2011 | 6 | 10 | 4 |
| Tu | Psychological Effects of False-Positive Results in Expanded Newborn Screening in China | 2012 | 4 | 10 | 4 |
| Ulph | Psychological and Ethical Challenges of Introducing Whole Genome Sequencing into Routine Newborn Screening: Lessons Learned from Existing Newborn Screening | 2022 | 1, 2, 3, 4 | 1, 2, 3, 7, 10 |  |
| Ulph | Familial influences on antenatal and newborn haemoglobinopathy screening | 2011 | 5 familly can support, 1, 2, 6 | 3, 6 | 4 |
| van der pal | Parents’ views on accepting, declining, and expanding newborn bloodspot screening | 2022 | 1, 5 | 3, 5, 6, 7, 9, 10 | 1, 7 |
| van Dijk | Expanding Neonatal Bloodspot Screening: A Multi-Stakeholder Perspective | 2021 | 1, 2, 3, 5 | 4, 5, 7 | 1,2,6 |
| Vansenne | Providing Genetic Risk Information to Parents of Newborns with Sickle Cell Trait: Role of the General Practitioner in Neonatal Screening | 2011 | 1, 2 | 3 | 4 |
| Vernooij-van Langen A | Parental knowledge reduces long term anxiety induced by false-positive test results after newborn screening for cystic fibrosis | 2014 | 6 | 1, 7, 10 | 3 |
| Wade | Young adults’ attitudes toward pediatric whole-genome sequencing | 2016 | 1, 3, 5 | 3, 6, 7, 10 | 1, 2 |
| Waisbren S | Psychosocial Factors Influencing Parental Interest in Genomic Sequencing of Newborns | 2015 | 6 | 7 | 7 |
| Walsh Lang C | Maternal Attitudes About Sickle Cell Trait Identification in Themselves and Their Infants | 2010 | 6 | 2, 3, 6, 10 | 1, 2, 7 |
| Wang | Current attitudes and preconceptions on newborn genetic screening in the Chinese reproductive‑aged population | 2022 | 1, 2, 5 | 3, 4, 5, 7, 10 | 2 |
| Weinreich S | Public support for neonatal screening for Pompe disease, a broad-phenotype condition | 2012 | 1, 3, 5 | 5, 7, 9 | 2, 7 |
| Wood M | PARENTAL ATTITUDES TOWARD NEWBORN SCREENING FOR DUCHENNE/BECKER MUSCULAR DYSTROPHY AND SPINAL MUSCULAR ATROPHY | 2014 | 1, 2 | 1, 4, 10 | 1, 7 |
| Wright S | Understanding Midwives’ Preferences for Providing Information About Newborn Bloodspot Screening | 2018 | 5 | 3, 7, 10 | 4 |
| Wright S | Eliciting Preferences for Information Provision in Newborn Bloodspot Screening Programs | 2017 | 6 | 3, 10 | 1, 2 |
| Wu | Are We Ready for Newborn Genetic Screening? A Cross-Sectional Survey of Healthcare Professionals in Southeast China | 2022 | 4 | 3, 4, 5, 6, 7, 10 | **1**, 5, **8** |
